# Genic non-coding microsatellites in the rice genome: characterization, marker design and use in assessing genetic and evolutionary relationships among domesticated groups

**DOI:** 10.1186/1471-2164-10-140

**Published:** 2009-03-31

**Authors:** Swarup Kumar Parida, Vivek Dalal, Ashok Kumar Singh, Nagendra Kumar Singh, Trilochan Mohapatra

**Affiliations:** 1 National Research Centre on Plant Biotechnology, Indian Agricultural Research Institute, New Delhi, 110012, India; 2 Division of Genetics, Indian Agricultural Research Institute, New Delhi, 110012, India

## Abstract

**Background:**

Completely sequenced plant genomes provide scope for designing a large number of microsatellite markers, which are useful in various aspects of crop breeding and genetic analysis. With the objective of developing genic but non-coding microsatellite (GNMS) markers for the rice (*Oryza sativa *L.) genome, we characterized the frequency and relative distribution of microsatellite repeat-motifs in 18,935 predicted protein coding genes including 14,308 putative promoter sequences.

**Results:**

We identified 19,555 perfect GNMS repeats with densities ranging from 306.7/Mb in chromosome 1 to 450/Mb in chromosome 12 with an average of 357.5 GNMS per Mb. The average microsatellite density was maximum in the 5' untranslated regions (UTRs) followed by those in introns, promoters, 3'UTRs and minimum in the coding sequences (CDS). Primers were designed for 17,966 (92%) GNMS repeats, including 4,288 (94%) hypervariable class I types, which were bin-mapped on the rice genome. The GNMS markers were most polymorphic in the intronic region (73.3%) followed by markers in the promoter region (53.3%) and least in the CDS (26.6%). The robust polymerase chain reaction (PCR) amplification efficiency and high polymorphic potential of GNMS markers over genic coding and random genomic microsatellite markers suggest their immediate use in efficient genotyping applications in rice. A set of these markers could assess genetic diversity and establish phylogenetic relationships among domesticated rice cultivar groups. We also demonstrated the usefulness of orthologous and paralogous conserved non-coding microsatellite (CNMS) markers, identified in the putative rice promoter sequences, for comparative physical mapping and understanding of evolutionary and gene regulatory complexities among rice and other members of the grass family. The divergence between long-grained aromatics and subspecies *japonica *was estimated to be more recent (0.004 Mya) compared to short-grained aromatics from *japonica *(0.006 Mya) and long-grained aromatics from subspecies *indica *(0.014 Mya).

**Conclusion:**

Our analyses showed that GNMS markers with their high polymorphic potential would be preferred candidate functional markers in various marker-based applications in rice genetics, genomics and breeding. The CNMS markers provided encouraging implications for their use in comparative genome mapping and understanding of evolutionary complexities in rice and other members of grass family.

## Background

Microsatellites or simple sequence repeats are tandemly repeated 1–6 base-pair (bp) nucleotide motifs distributed across the genome in many prokaryotes and eukaryotes [1]. An increasing number of microsatellites have been characterized in protein coding sequences (CDSs) and non-coding untranslated regions (UTRs) of genes for several plant species. Alterations in these microsatellite sequences are thought to have significant consequences with regard to gene function [2]. Variation in the length of microsatellite motifs in non-coding sequences of genes (i.e. promoters, UTRs and introns) may affect the process of transcription and translation through slippage, gene silencing and pre-mRNA splicing as has been observed for many diseases in humans, including cancers and neuronal disorders [3-9]. Microsatellite markers based on such sequence motifs would be useful as "functional genetic markers" for various applications in genomics and crop breeding. However, the identification and characterization of such microsatellites has been limited in plants.

Completely sequenced genomes provide scope for designing a large number of gene based microsatellite markers. Rice (*Oryza sativa *L.) is the first cereal with a completely sequenced genome that has enabled the development of a large number of microsatellite markers [10]. Recently, Zhang *et al*. [11] developed 52,485 microsatellite markers polymorphic between *indica *and *japonica*. It is difficult to choose useful and informative microsatellite markers from large marker data-sets for genotyping applications in rice. This can be overcome by constructing a smaller informative microsatellite marker database comprising markers located in potentially functional genic sequences with relatively high polymorphic potential. However, genic microsatellite markers when derived from protein-coding sequences are constrained by purifying selection [12] and thus have less potential for revealing polymorphism particularly at the intra-specific level [13]. In contrast, markers derived from non-coding sequence components (i.e. 5'UTRs, introns and 3'UTRs) are under moderate selection pressure and thus expected to be more polymorphic as genetic markers. Previous studies have shown non-random and distinct patterns of microsatellite distribution in non-coding sequence components of rice genes predicted in the completely sequenced rice genome [14]. In view of the excellent genetic attributes and higher informativeness expected for genic non-coding microsatellite (GNMS) markers, development of such markers from the protein coding genes predicted in the rice genome would be of practical significance.

A comparative analysis of non-coding sequences, known as phylogenetic footprinting [15-19], has provided useful inferences about conserved non-coding microsatellite (CNMS) repeat-containing regulatory sequence elements and their significance in gene regulation in plant-specific pathways [20]. These studies have suggested the use of completely finished and recently annotated rice genomic sequences for intra- and inter-genomic phylogenetic footprinting to detect a large number of paralogous and orthologous CNMS motifs, respectively, in the 5' non-coding promoter regions of genes among cereals and *Arabidopsis thaliana*. Identification of such CNMS motifs would help in understanding the pattern of regulatory or non-coding promoter sequence evolution in plant genomes.

We undertook this study to characterize the frequency and relative distribution of GNMS repeat-motifs in different sequence components of protein coding rice genes; design primers flanking the GNMS repeat-motifs; physically locate the markers on rice chromosomes, and evaluate their efficiency in the assessment of molecular diversity; detect and characterize CNMS motifs in the putative promoter regions of rice genes using intra- and inter-genomic phylogenetic footprinting; and evaluate markers for their utility in comparative physical mapping and establishing molecular phylogenetic relationships among different rice cultivar groups.

## Results and discussion

### Frequency and relative abundance of GNMS

We identified 19,555 perfect GNMS (excluding mononucleotides) in 18,935 protein coding genes including 14,308 putative promoter sequences predicted in the rice genome. The density of perfect GNMS varied from 306.7/Mb in chromosome 1 to 450/Mb in chromosome 12 with an average of 356.7 GNMS/Mb (Table 1). This included 4,657 (32.5%) GNMS repeats in putative promoters, 4,843 (25.6%) in 5'UTRs, 8,996 (12.8%) in introns and 1,020 (5.4%) in 3'UTRs (Table 1). Of the promoter-derived GNMS repeats, 342 (16.6%) were found in 2,060 TATA box containing promoter sequences. The average density of compound GNMS in the rice genes was 78.3/Mb with maximum density (99.8/Mb) in chromosome 12 and minimum density (68.5/Mb) in chromosome 4. The perfect GNMS density in the promoters was maximum (388/Mb) in chromosome 3 and minimum (249.5/Mb) in chromosome 11, whereas in the 5'UTRs it varied from 816.1/Mb in chromosome 11 to 1360/Mb in chromosome 8 (see Additional file 1). The intronic GNMS density was maximum (514.5/Mb) in chromosome 11 and minimum (237/Mb) in chromosome 3, while in the 3'UTRs the GNMS density ranged from 96/Mb in chromosome 4 to 168.3/Mb in chromosome 12. The average microsatellite density was maximum (1144.8/Mb) in the 5'UTRs followed by introns (327.3/Mb), promoters (300.8/Mb) and 3'UTRs (135.9/Mb) compared to 182.4/Mb in the CDS (Table 1). Thus, the overall GNMS density increased gradually from the upstream of the transcription start sites (TSS) and reached a peak (about 3.8 times higher than the GNMS density in the promoters) in the downstream of the TSS (i.e. 5'UTRs). Subsequently, the density decreased and dropped down in the coding region and showed asymmetrical distribution along the direction of transcription. However, this asymmetry arose because of high GNMS density in the introns; about 1.8 and 2.4 times higher than that in the CDS and 3'UTRs, respectively. Our results are in contrast to an earlier report from Fujimori *et al*. [21], which suggested gradual lowering of microsatellite density along the direction of transcription based on the analyses of 28,469 full-length cDNA sequences. This may be because we used annotated rice gene sets having UTRs, introns and promoter sequences predicted from the completely sequenced rice genome that provided a better evaluation of GNMS density in the different sequence components studied. The most frequent occurrence of GNMS motifs in the 5'UTRs is consistent with earlier observations in rice and *Arabidopsis *genomes [14]. Interestingly, the majority (57.6%) of the GNMS motifs in the 5'UTRs were present in various transcription- and translation-related genes encoding transcription/translation initiation and elongation factors and ribosomal proteins. This suggests a functional significance of the repeat motifs present in the 5' UTRs, which needs to be further investigated.

**Table 1:** Nature, frequency and relative distribution of microsatellites in the four non-coding and coding sequence components of rice genes distributed over 12 chromosomes

**Characters under study**	**Putative Promoters**	**5'UTRs**	**Introns**	**3'UTRs**	**Total GNMS**	**Range of values observed for individual rice chromosomes**	**CDS**
Number of sequences examined	14308	18935	70083	18935	122261	5799 (Chr 10) to 18954 (Chr 1)	18935
Size (bp) of examined sequences	15481256	4230243	27485418	7506247	54703164	2786342 (Chr 9) to 8429186 (Chr 1)	25733724
Number (%) of identified perfect microsatellites	8366 (58.5)	5780 (30.5)	25495 (36.4)	2817 (14.8)	42458	5920 (31.2, Chr 1) to 2475 (42.4, Chr 11)	4828 (25.5)
Number (%) of mononucleotides	3709 (26)	937 (4.9)	16499 (23.5)	1797 (9.5)	22942	2263 (12, Chr 1) to 1348 (23, Chr 11)	134 (0.7)
Number (%) of dinucleotides	2187 (46.9)	1508 (31)	5805 (64.5)	507 (49.7)	10007	1299 (21.9, Chr 1) to 742 (27.6, Chr 12)	139 (3)
Number (%) of trinucleotides	2351 (50.5)	3171 (65.5)	2586 (28.7)	437 (42.8)	8545	298 (12.8, Chr 10) to 625 (21.5, Chr 8)	4514 (96.2)
Number (%) of tetranucleotides	82 (1.7)	100 (2)	494 (5.5)	68 (6.7)	744	38 (1, Chr 4) to 70 (2.4, Chr 8)	21 (0.4)
Number (%) of pentanucleotides	34 (0.7)	52 (1.1)	81 (0.9)	8 (0.8)	175	9 (0.24, Chr 4) to 16 (0.59, Chr 12)	10 (0.2)
Number (%) of hexanucleotides	3 (0.06)	12 (0.25)	30 (0.33)	1 (0.09)	46	1 (0.04, Chr 1) to 10 (0.2, Chr 2)	10 (0.2)
Number (%) of perfect microsatellites excluding mononucleotides	4657 (32.5)	4843 (25.6)	8996 (12.8)	1020 (5.4)	19516 (16)	1087 (18, Chr 9) to 2585 (13.6, Chr 1)	4694 (24.8)
Number (%) of primer-pairs for perfect microsatellites	4278 (91.8)	4484 (92.6)	8370 (93)	834 (81.7)	17966 (92)	938 (86.3, Chr 9) to 2395 (92.6, Chr 1)	4411 (94)
Perfect microsatellite counts per Mb sequences	300.8	1144.8	327.3	135.9	356.7	450 (Chr 12) to 306.7 (Chr 1)	182.4
Number (%) of compound microsatellites	948 (6.6)	746 (3.9)	2421 (3.4)	169 (0.9)	4284 (3.5)	217 (3.6, Chr 9) to 585 (3, Chr 1)	510 (2.7)
Compound microsatellite counts per Mb sequences	61.2	176.3	88.0	22.5	78.3	99.8 (Chr 12) to 68.5 (Chr 4)	19.8
Number (%) of perfect class I microsatellites	877 (18.8)	1031 (21.3)	2394 (26.6)	257 (25.2)	4559 (23.3)	229 (21, Chr 10) to 598 (24.4, Chr 3)	743 (15.8)
Number (%) of primer-pairs for perfect class I microsatellites	829 (94.5)	953 (92.4)	2275 (95)	231 (89.8)	4288 (94)	216 (94.3, Chr 10) to 571 (95.5, Chr 3)	714 (96%)
Perfect class I microsatellite counts per Mb sequences	56.6	243.7	87.1	34.2	83.8	101.4 (Chr 12) to 67.7 (Chr 1)	28.8

### Nature and distribution of GNMS

The GC-rich trinucleotide GNMS repeat-motifs were the most prevalent class of microsatellites in the regulatory regions (i.e. 5'UTRs and promoters), whereas the AT-rich trinucleotide repeats were distributed evenly in all the coding and non-coding sequence components. However, the proportion of GC-rich trinucleotide motifs was maximum in the 5'UTRs (65.5%) followed by putative promoters (50.5%), 3'UTRs (42.8%) and introns (28.7%) compared to 96.2% in the CDS (Table 1, see Additional file 2). This trend corresponds to GC-rich microsatellites being frequently detected in the regions downstream of TSS possibly due to higher GC content at the 5'end of the rice genes [22]. The GC-rich trinucleotide GNMS repeat-motifs in the 5'UTRs and promoters perhaps serve as binding sites for nuclear proteins that are essential for regulating translation and gene expression, and thus are expected to occur more frequently in these sequences [23]. The high frequency of trinucleotide GNMS repeat-motifs in the coding regions could be due to selection against frameshift mutations that limits expansion of non-triplet microsatellites [24]. These results agreed well with earlier observations on the relative abundance of GC-rich trinucleotide repeat-motifs in the expressed sequence tags and unigene sequences of cereal genomes [25,26]. The dinucleotide and tetranucleotide repeat-motifs were predominant particularly in the intronic and 3'UTR sequences (see Additional file 2). The AT-rich dinucleotide repeat-motifs were most in intronic sequences (64.5%) followed by 3'UTRs (49.7%), whereas the proportion of AT-rich tetranucleotide repeats was maximum in 3'UTRs (6.7%; Table 1). The purine-rich dinucleotide microsatellites, such as (GA)n, were abundant in 5'UTRs (28.6%) followed by promoters (18.4%) compared to 28% in CDS (see Additional file 1). Our observations are comparable to those from earlier studies on abundance of GA-rich dinucleotide repeat-motifs in the coding [25,26] and 5'-end flanking regions [14] and AT-rich dinucleotide motifs in the intronic sequences in rice genes [27]. The promoter sequences of rice genes frequently (22.3%) contained pyrimidine-rich microsatellites, especially (CT)n dinucleotides (see Additional file 1), possibly due to their potential role in activation of promoters for transcription initiation [28].

The microsatellite with longer repeat-motifs is expected to be more polymorphic due to high length dependent replication slippage [27]. We identified 4,559 class I GNMS repeat-motifs in the protein coding genes predicted in the rice genome with an overall density of 83.8 GNMS/Mb (Table 1). The density of the class I repeat-motif containing GNMS varied from 67.7/Mb in chromosome 1 to 101.4/Mb in chromosome 12 whereas its proportion ranged from 18.8% (877) in promoters to 26.6% (2394) in intronic sequences (Table 1). Thus, the potential of microsatellite expansion in the genic non-coding sequences of rice genes is not correlated with the frequency of GC-rich trinucleotide repeat-motifs in these regions [27]. Our results revealed non-random and strongly biased distribution of GNMS repeat-motifs across the regulatory and non-coding regions of the rice genes.

### Design and physical location of GNMS markers

Forward and reverse primers were designed from the flanking sequences of the identified microsatellite repeat-motifs in each of the four genic non-coding sequence components of rice genes (i.e. promoters, 5'UTRs, introns and 3'UTRs). The structural organization of the various sequence components and their implications for designing different types of GNMS markers are shown in additional file 3. Primers were designed for 17,966 (92%) genic non-coding sequences – 4,278 (91.8%) in promoters, 4,484 (92.6%) in 5'UTRs, 8,370 (93%) in introns and 834 (81.7%) in 3'UTRs. The primer sequences for 4,288 (94%) hypervariable class I GNMS markers distributed across 12 rice chromosomes are provided in additional file 4 (sequences for the remaining primer-pairs are available on request). Class I markers included 829 in promoters, 953 in 5'UTRs, 2,275 in introns and 231 in 3'UTRs. The GNMS markers were present in the rice genes that regulate biological and cellular functions. For example, we identified 51 (7 in promoters, 12 in 5'UTRs, 27 in introns, 5 in 3'UTRs) GNMS markers including 10 class I types in various disease resistance genes predicted in rice chromosome 11 (see Additional file 3) and 25 (4 in promoters, 9 in 5'UTRs, 11 in introns, 1 in 3'UTRs) GNMS markers including 7 class I types in rice chromosome 12 (see Additional file 4). The GNMS markers when genetically associated with the target traits would facilitate gene cloning and marker-assisted breeding, thereby accelerating rice genetic improvement.

**Figure 1:**
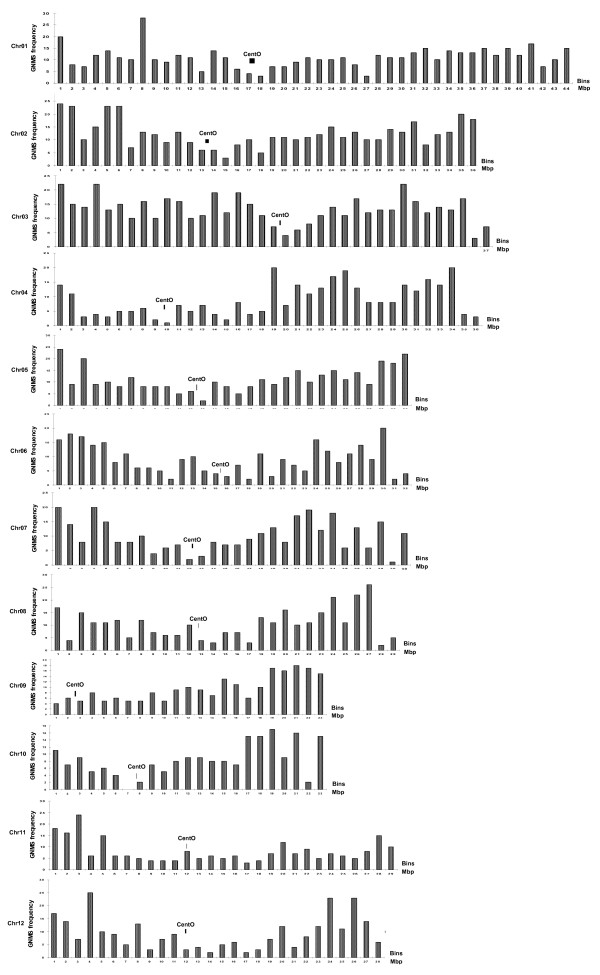
**Physical distribution of bin-mapped GNMS markers on rice chromosomes**. The distribution frequency of 4,288 class I mapped GNMS markers on 12 rice chromosomes. The frequency corresponds to number of GNMS markers mapped per 1 Mb sized bins. In general, the mapped GNMS markers showed more concentration on both the arms and towards the telomeric ends of all rice chromosomes compared to those of centromeric regions except for chromosomes 4, 9 and 10, which are known to be highly heterochromatic.

We determined the distribution of class I GNMS markers designed from the 4 genic non-coding sequence components based on their physical location (bp) on rice chromosomes. We divided each rice chromosome into 1 Mb interval sized physical bins and integrated 4,288 class I GNMS markers present in 3,874 rice genes based on their ascending order of physical location (bp) beginning from the short arm telomere to the long arm telomere (see Additional file 5). Detailed information regarding the physical position of the various GNMS markers on the rice chromosomes is provided in additional file 4. The map density of class I GNMS markers varied from 126 kb (226) in chromosome 11 to 74 kb (487) in chromosome 3 with an average of 100.7 kb (see Additional file 6). The maximum map density in rice chromosome 3 could be due to its greater physical size, maximum gene density and least transposon association compared to other rice chromosomes [10]. In general, mapped GNMS markers showed more concentration on both arms and towards the telomeric ends of all rice chromosomes than in the centromeric regions except for chromosomes 4, 9 and 10 (Figure 1). This possibly showed correspondence with the higher density of genes on the chromosome arms/telomeric regions of most of the rice chromosomes than in the centromeres [10,29]. A maximum of 20 and a minimum of 2 markers were present per 1 Mb physical bin of rice chromosome (Figure 1). The low number of markers in some bins could be due to either the absence of euchromatin sequences or least class I microsatellite containing rice genes at these intervals. The GNMS marker based rice physical map would provide invaluable information on the genomic distribution of these markers and thus aid marker selection for many applications in rice breeding and genomics.

### Amplification efficiency and polymorphic potential of GNMS markers

We used 15 microsatellite markers designed (see Additional file 7) from each of the 4 genic non-coding sequence components as well as from CDS of rice genes to understand their potential to amplify the target sequence and detect polymorphism among a set of 18 rice genotypes (7 non-aromatic *indica *genotypes, 6 long-grained traditional and elite Basmati cultivars, 3 short-grained aromatics and 2 *japonica *genotypes). Fifty-six of the 60 markers gave amplification around 55°C annealing temperature with a success rate of 93.3% that suggested the utility of genic non-coding sequences (with balanced GC content of 48% to 52%) for developing large-scale microsatellite markers in rice. The remaining 4 primers (6.7%), which were derived from the intronic sequences, did not show amplification in any of the non-aromatic *indica *and Basmati rice genotypes. However, amplification was observed for these markers in the *japonica *genotypes from which sequence the primers were designed. The occurrence of null alleles could be due to insertion/deletions (InDels) in the corresponding genomic sequences of *indica *and *japonica *[30,31]. It may also be the result of frequent association of intronic microsatellites with the micropon family of miniature inverted-repeat transposable elements (MITES) in rice [27]. Thirty (53.6%) of the 56 GNMS markers that underwent amplification showed polymorphism (see Additional file 3). The number of alleles amplified per locus varied from 2 to 8 (Table 2). Thirty polymorphic GNMS markers amplified 130 alleles with a mean allele number of 4.33. Most (80%) of the GNMS markers showing polymorphism were class I type repeat-motifs. The polymorphism information content (PIC) value ranged from 0.20 to 0.86 with an average of 0.66 (Table 2). The extent of polymorphism detected by the GNMS markers is comparable to that reported previously with random genomic microsatellite markers in a set of rice genotypes [32-34].

**Table 2:** Comparative evaluation of polymorphic potential of the microsatellite markers designed from the non-coding and coding sequence components of rice genes

				**Number of alleles**			
					
**Sequence components of genes**	**GNMS markers**	**Number of GNMS markers amplified**	**Number (%) polymorphic GNMS markers**	**Minimum**	**Maximum**	**Average**	**Mean PIC**
Promoters	15	15	8 (53.3)	2	6	4	0.64
5'UTRs	15	15	6 (40)	2	5	3.5	0.62
Introns	15	11	11 (73.3)	2	8	5	0.72
3'UTRs	15	15	5 (33.3)	2	4	3	0.58
**Total GNMS**	**60**	**56**	**30 (53.5)**	**-**	**-**	**-**	**0.64**
CDS	15	15	4 (26.6)	1	2	1.5	0.10

We detected polymorphism with 11 (73.3%, PIC of 0.72) markers from intronic sequences, 8 from promoters (53.3%, PIC of 0.64), 6 from 5'UTRs (40%, 0.62), 5 from 3'UTRs (33.3%, 0.58) and 4 from CDS (26.6%, 0.10) (Table 2). Among the intronic GNMS markers, the one based on the ubiquitin gene (see Additional file 8) showed the maximum PIC value (0.86) followed by that on ribosomal protein S35 (0.84). The higher level of polymorphism we observed for the GNMS markers derived from the promoters, UTRs and introns are expected due to the presence of the most abundant and polymorphic class of GA- or AT-rich dinucleotide microsatellite repeat-motifs in these sequence components. Further, a comparative evaluation of the polymorphic potential of 225 GNMS markers distributed over 12 rice chromosomes with that of 600 rice microsatellite (RM) [35] series markers in two parental genotypes (Jaya and NPT-11) of a large mapping population revealed higher efficiency for GNMS markers (32%) over RM markers (19%) in detecting parental polymorphism (unpublished results). The GNMS markers, being more informative than the genic coding and random genomic microsatellite markers developed earlier would be of immediate use in efficient large-scale genotyping applications in rice [36]. Twenty-six (46.4%) of the 56 GNMS markers showed polymorphism between the *indica *and *japonica *genotypes, while 22 (39.3%) revealed polymorphism among the *indica *genotypes. GNMS markers derived from the intronic sequences showed maximum inter sub-specific polymorphism as reported for intron length polymorphisms in rice [37]. Sequencing of the amplicons obtained with the 8 GNMS markers, from the genic non-coding and coding sequence components that showed amplification for all the 18 rice genotypes, confirmed the presence of target repeat motifs (see Additional file 9).

### Assessment of molecular genetic diversity among domesticated rice cultivar groups

**Figure 2:**
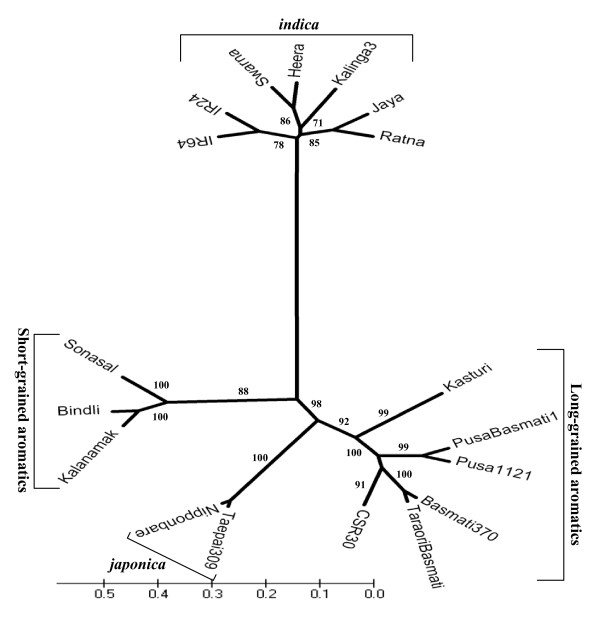
**Phylogenetic tree depicting genetic relationships among domesticated rice genotypes**. Unrooted phylogenetic tree depicting the molecular genetic relationships among non-aromatic *indica*, long-grained traditional and evolved Basmati, short-grained aromatics and temperate and tropical *japonica *rice genotypes based on Nei and Li's similarity coefficient using 56 GNMS markers. Percentages of confidence obtained in bootstrap analysis are indicated at the corresponding node for each cluster. Molecular classification corresponded to known evolutionary relationship and phenotypic classification.

The pair-wise similarity index among the 18 rice genotypes, based on the combined profiles of all the 56 GNMS markers, revealed a broad range from 0.15 to 0.74 with an average of 0.33 (see Additional file 10). This level of diversity is much higher than that detected previously (0.32 to 0.58 with an average of 0.44 [33], 0.45 to 0.61 with an average of 0.39 [34]) with random genomic microsatellite markers. Our result indicates the greater efficiency of GNMS markers, which assayed potentially functional genetic diversity in the rice genome. The aromatic group (including long- and short-grained aromatics) had a relatively higher average similarity (0.24) with *japonica *compared to *indica *genotypes (0.13). The results of higher evolutionary closeness between *japonica *and aromatics are consistent with earlier nuclear and chloroplast diversity studies based on microsatellite [34] and single nucleotide polymorphism [38] markers. The long-grained aromatic cultivars were most divergent as reflected by their broader similarity index (range: 0.29 to 0.58, average 0.42). This could be due to the inclusion of both traditional and improved Basmati cultivars in this group. The higher level of diversity among long-grained aromatics further agreed well with our observations of higher proportion of polymorphic loci in these cultivars. The relationship among the 18 rice genotypes is depicted in an unrooted phylogenetic tree (Figure 2). It revealed two distinct clusters: one comprising long-grained traditional and elite Basmati, short-grained aromatics and *japonica *genotypes; and another comprising only *indica *genotypes. The GNMS marker based clustering clearly differentiated all 18 rice genotypes from each other and resulted in a definitive grouping with high bootstrap values (71 to 100) that corresponded well with their known phenotypic classification and evolutionary relationships [32-34]. These GNMS markers could be used for establishing distinctness among rice varieties.

### Evolutionary significance of CNMS containing rice promoters

Using inter-genomic phylogenetic footprinting we detected 112 CNMS repeats (14.6% of the 767 microsatellites containing promoter sequences of rice genes) in the putative promoter sequences of orthologous genes among 5 cereal genomes (viz. barley, maize, rice, *Sorghum*, wheat; see Additional file 11) and 67 (8.7%) CNMS repeats in the promoters of orthologous genes of rice and *A. thaliana *(see Additional file 12). With intra-genomic phylogenetic footprinting we identified 45 (5.9%) CNMS markers in the promoters of paralogous rice genes (see Additional file 13). The CNMS markers identified among the 5 cereal genomes included 43 in the promoters between orthologous rice and maize genes, 26 between rice and barley, 28 between rice and wheat and 15 between rice and *Sorghum *(see Additional file 11). Results from intra- and inter-genomic phylogenetic footprinting comparisons showed that 11 CNMS motifs were conserved in the promoters of both orthologous and paralogous rice genes (see Additional file 14). The CT-rich dinucleotide repeat-motifs were the predominant microsatellite classes in the CNMS, which is consistent with the characteristics of pyrimidine-rich repeat distribution in the promoter regions of the rice genes as observed in our study. A low frequency of CNMS motifs indicated a relatively rapid evolution of rice promoters having such sequences, which could be due to functional constraints and rapid adaptive changes in the regulatory regions of homologous genes for imparting specific roles in gene regulation. This may be why most of the identified CNMS repeats were located in the orthologous and paralogous genes in the immediate upstream region (1 to 200 bp) of the transcription initiation site with preference for known regulatory binding sites as characterized by PLACE and PlantCARE (see Additional file 15). It is possible that these sequences are involved in gene regulation in response to environmental stimuli [21]. For example, the CNMS motif (GA)n in the promoters of rice gene cytochrome P450, contained sequences similar to GAGA (AGAGAGAGA), a known regulatory element, which is involved in light responsive phototransduction regulation in plants. Complementary to (GA)n, the CNMS motif (CT)n contained a different regulatory element, C2C2-GATA (TCTCTCTCTCT), controlling similar light responsive gene regulation in the serine/threonine protein kinase gene. A comparative physical mapping of CNMS markers of rice chromosome 1 and homeologous chromosomes of 4 other cereal species (barley, maize, *Sorghum*, wheat) and one dicot species (*A. thaliana*) detected several collinear regions with complex chromosomal syntenic relationships (see Additional file 16), which provide clues to the role of identified CNMS markers in comparative genomics and in the understanding of evolutionary complexities in cereals and *Arabidopsis*.

Modal nucleotide substitutions for the 11 orthologous and paralogous rice CNMS containing promoters indicated that barley, maize, *Sorghum *and wheat diverged from rice after the rice genomic duplication events (72.4 Mya) and monocot-dicot speciation (124.7 Mya) (Table 3). The divergence time between rice and each of the 4 cereal species was consistent with the separation time (about 50 Mya) of cereals [39], of which maize (22 Mya) has diverged more recently (Table 3). An analysis of modal nucleotide substitutions in promoter regions of 8 sequenced CNMS loci among the domesticated rice cultivar groups indicated high modal nucleotide substitutions (0.0010) and thus wider divergence between *indica *and *japonica *rice from a common ancestor at 0.40 Mya (Table 3). The divergence between long-grained aromatics and *japonica *was more recent (modal substitution 0.000011, 0.004 Mya) compared to short-grained aromatics from *japonica *(0.000017, 0.006 Mya) and long-grained aromatics from *indica *(0.000033, 0.014 Mya). Among the aromatics, the divergence of short- and long-grained types (0.000029, 0.012 Mya) was in-between the separation time of short- and long-grained aromatics from *indica *(Table 3). Our results thus supported the earlier observations about the origin of *indica *and *japonica *from an ancestral rice genotype [40] before their domestication about 10,000 to 12,000 years ago [41,42], and the evolutionary closeness between *japonica *and aromatic varieties [34]. Our results from molecular dating of divergence showed that *indica *is most diverged from others and that aromatics are an intermediate group between *indica *and *japonica *subspecies; much closer, however, to *japonica *than *indica*. The higher divergence time between short- and long-grained aromatics than between each of them and *japonica *was suggestive of the higher allelic diversity within the aromatics than in *indica *and *japonica *rice cultivars. However, earlier studies have observed lower genetic diversity in aromatic rice relative to other cultivar groups [33,34]. This contrasting result is due to inclusion of poor-yielding traditional Basmati varieties, which are the products of selection from land-races and improved high-yielding Basmati varieties developed through cross-breeding involving traditional Basmati and non-Basmati genotypes in the aromatic group in our study.

**Table 3:** Intra- and inter-specific molecular dating of divergence of CNMS containing rice promoter sequences

**Species/domesticated rice cultivar groups**	**No. of paralogous/orthologous CNMS**	**Percent CNMS**	**Modal substitutions**	**Duplication event/time of divergence (Mya)**
Rice from rice	45	6.3	0.180	72.4
Wheat from rice	28	3.5	0.067	27.0
Maize from rice	43	5.6	0.054	22.0
*Sorghum *from rice	15	3.7	0.082	33.0
Barley from rice	26	1.9	0.097	39.0
*Arabidopsis *from rice	67	1.2	0.310	124.7
*indica *vs. *japonica*	8	3.1	0.0010	0.40
*indica *vs. short-grained aromatics	8	3.1	0.000027	0.010
*indica *vs. long-grained aromatics	8	3.0	0.000033	0.014
Short-grained vs. long-grained aromatics	8	3.1	0.000029	0.012
Short-grained aromatics vs. *japonica*	8	3.2	0.000017	0.006
Long-grained aromatics vs. *japonica*	8	3.6	0.000011	0.004

## Conclusion

We studied relative distribution of microsatellites in different sequence components of protein coding rice genes, designed 17,966 GNMS markers, including 4,288 hypervariable class I types from the promoter, 5'UTR, intronic and 3'UTR sequences and determined their occurrence and organization on the 12 rice chromosomes. The class I markers were bin-mapped to guide the selection of markers with genome wide distribution for various genotyping applications in rice. We demonstrated the utility of GNMS markers by their robust PCR amplification efficiency and high potential for detecting polymorphism over genic coding and random genomic microsatellite markers, and thus their immediate use in rice genetics, genomics and breeding. The unrooted phylogenetic tree constructed based on molecular diversity values of a set of GNMS markers in rice genotypes clearly established molecular genetic relationships among the domesticated rice cultivar groups, thereby suggesting their utility in defining varietal identity in commerce. The orthologous and paralogous CNMS markers identified in the rice promoters would be useful for comparative genome mapping and phylogenetic analysis in rice and other members of grass family

## Methods

### Accessing the genic non-coding sequences of the rice genome

The latest annotated 28,763 non-transposable element (TE)-related rice genes (individually for each of the 12 rice chromosomes) were acquired in FASTA format from the TIGR rice genome annotation database release 5.0 (24^th ^Jan' 2007) using an ftp server [43]. Of these, 25,447 genes were found to contain defined UTR sequences. A set of 6,512 of the 25,447 rice genes identified to have alternatively spliced isoforms were excluded from our analysis. To determine the density of microsatellites accurately, we screened 18,935 rice gene models representing only one splice form with defined UTRs, CDS and introns for further analyses.

### Identification and characterization of promoter sequences

For identifying and characterizing the putative promoter sequences, we assessed the genomic FASTA sequences 1000 bp upstream of the transcription start site chromosome-wise individually for 18,935 rice genes and used the TSSP SoftBerry plant promoter prediction program [44]. The results from 16,738 rice genes containing defined promoter sequences with a description of putative *cis*-regulatory elements were stored separately for the 12 rice chromosomes. These predicted promoter sequences were BLAST searched against the annotated 13,046 rice eukaryotic promoter database (EPD) [45] chromosome-wise and compared with major databases namely, PLACE [46] and PlantCARE [47] for the identification of transcription factor binding sites and *cis*-regulatory elements. Based on the BLAST results (with matching *E *value = 0 and bit score ≥ 500), 14,308 robust promoter sequences were finally identified in the whole rice genome for further analyses.

### Mining of microsatellites and primer design

The genic non-coding sequences of 18,935 rice genes including 14,308 putative promoter sequences were searched for microsatellites as described earlier [26] and compared with those with the CDS in each of the 12 rice chromosomes. The nature, frequency and relative abundance of various repeat-motif classes including hypervariable class I (≥ 20 nucleotides) and potentially variable class II (12 to 20 nucleotides) types were determined individually for promoters, 5'UTRs, CDS, introns and 3'UTRs of the rice genes. We designed primers from the flanking sequences of the identified repeat-motifs in each of these 5 sequence components of rice genes as described earlier [26].

### Distribution of GNMS markers in the rice genome

The specific physical location of class I GNMS markers designed from the promoters, 5'UTRs, introns and 3'UTRs of rice genes was determined based on their annotated physical positions (bp) on the rice chromosomes provided in the latest released TIGR rice pseudomolecule 5.0 database. Individual rice chromosomes were divided into 1 Mb interval sized bins and the class I GNMS markers were plotted separately for each of the 12 rice chromosomes according to their ascending order of physical location (bp).

### Evaluation of amplification efficiency and polymorphic potential

The potential of GNMS markers to amplify the target sequence and detect polymorphism was evaluated using 15 markers we designed from the flanking sequences from each of the 5 sequence components (promoters, 5'UTRs, introns, 3'UTRs and CDS) of the rice genes. Genomic DNA was isolated from a set of 18 diverse rice genotypes (see Additional file 17) – 7 *indica*, 9 aromatic and 2 *japonica *rice genotypes – and used in PCR to amplify 75 GNMS markers. The amplified fragments were resolved in 10% native polyacrylamide gel using 0.5× TBE buffer (4 h at 220 V) and visualized under UV light after staining with GelStar (CAMBREX BioScience, USA). We used allelic data to estimate the number, range and distribution of amplified alleles, average polymorphic alleles per primer, percent polymorphism and PIC for all the amplified GNMS markers. The PIC value was calculated using the formula, PIC = 1 - ∑P_ij_^2 ^[48], where P_ij _is the frequency of the j^th ^allele for the i^th ^locus summed across all alleles for the locus. Cluster analysis among the 18 rice genotypes was based on Nei and Li similarity coefficient [49] by using the un-weighted pair group method analysis (UPGMA) in TREECON [50] software package. We determined the confidence limit of clusters by 500 bootstrap-replicates and constructed an unrooted phylogenetic tree by bootstrap of 50% majority rule consensus. To confirm that the GNMS markers did amplify the expected microsatellite repeat-motifs, 8 markers from each of the promoter, 5'UTR, intron, 3'UTR and CDS regions of rice genes that amplified in all the 18 rice genotypes were purified and sequenced. The high quality sequences were aligned and further examined for the presence of predicted repeat motifs.

### Detection and characterization of CNMS containing rice promoter sequences through intra- and inter-genomic phylogenetic footprinting

The predicted microsatellite containing promoter sequences of rice were BLAST searched against each other and with the 5' non-coding sequence regions of genes/expressed sequence tags annotated on completely sequenced bacterial artificial chromosomes anchored on the chromosomes and/bins of maize [51], *Sorghum *[52], wheat [53], barley [54] and *Arabidopsis *[55]. The matching sequences were aligned using a VISTA sequence alignment algorithm program [56,57] for identification and characterization of paralogous and orthologous CNMS containing promoters. A minimum percent nucleotide identity threshold of 70% and 20 bp as a minimal length criterion were considered significant in VISTA [18] for our analyses. The matching orthologous and paralogous CNMS containing rice promoter sequences were further characterized for known functional promoter regulatory elements using PLACE and PlantCARE software tools. The candidate CNMS containing rice promoters for cereal and *A. thaliana *genomes were identified. For comparative physical mapping, the physical positions (bp) of putative CNMS motifs on rice chromosome 1 (that carried more CNMS than other chromosomes) were determined and their physical order compared with that on homeologous chromosomes of 4 other cereals and *A. thaliana*.

### Estimation of intra- and inter-specific CNMS divergence

The CNMS containing promoter sequences of orthologous and paralogous rice genes were polled into alignments of 100–200 bp on average and used as inputs in the *baseml *program within the PAML version of PAL2NAL software package [58] for estimating nucleotide substitution rates among the CNMS sequences of cereals and *A. thaliana*. For estimating substitution rates among the *indica *and *japonica *cultivar groups, the CNMS repeat-motifs containing high quality promoter sequences of 8 rice genes that amplified in all the 18 rice genotypes were analyzed as described above. The modal nucleotide substitution obtained for the CNMS containing rice promoter sequences were used to estimate time (T) since divergence among the 5 cereals and *indica *and *japonica *cultivar groups as T (Mya) = Ks/2λ, where λ = mean rate of synonymous substitutions equal to 1.243 synonymous substitutions per 10^9 ^years [59].

## Authors' contributions

SKP conducted GNMS marker detection, marker design, physical mapping, polymorphism survey, diversity estimation, CNMS repeat characterization, expression analysis and drafted the manuscript. AKS was associated with the survey of polymorphism among the rice genotypes. VD participated in the acquisition of the database and *in silico *data analysis. NKS helped in data analyses and drafting of the manuscript. TM designed the study, guided data analysis and interpretation, participated in drafting and correcting the manuscript critically and gave the final approval of the version to be published. All authors have read and approved the final manuscript.

## Supplementary Material

Additional file 1 **Nature, frequency and relative distribution of microsatellites in the genic non-coding and coding sequences of the rice genome.** Click here for file

Additional file 2 **Frequency and abundance of various microsatellite repeat-motif classes in the genic non-coding and coding sequences of the rice genome.** Click here for file

Additional file 3 **Development of rice GNMS markers and their efficiency in detecting polymorphism.** Click here for file

Additional file 4 **Summary of 4,288 class I GNMS markers distributed on 3,874 rice genes with their physical positions on the 12 rice chromosomes and putative functions.** Click here for file

Additional file 5 **GNMS marker based physical bin map of rice genome.** Click here for file

Additional file 6 **Distribution and map-density of physically mapped class I GNMS markers on the 12 rice chromosomes.** Click here for file

Additional file 7 **Sixty GNMS markers and 15 microsatellite markers designed from the CDS used for evaluating their polymorphic potential among 18 rice genotypes.** Click here for file

Additional file 8 **Origin, distribution and polymorphic potential of 30 GNMS markers and four markers from the CDS, in a set of 18 rice genotypes.** Click here for file

Additional file 9 **Alignment showing the presence of class I GNMS repeat-motifs across 18 genotypes of rice.** Click here for file

Additional file 10 **Genetic relationships among 18 *indica *and *japonica *domesticated rice cultivars revealed by 56 rice GNMS markers.** Click here for file

Additional file 11 **Orthologous CNMS containing rice promoter sequences conserved among five cereal species.** Click here for file

Additional file 12 **Orthologous CNMS containing rice promoter sequences conserved in *Arabidopsis thaliana*.** Click here for file

Additional file 13 **CNMS containing promoter sequences identified in paralogous rice genes.** Click here for file

Additional file 14 **CNMS conserved in both orthologous and paralogous rice genes.** Click here for file

Additional file 15 **Positional distribution of orthologous and paralogous CNMS in promoter sequences of rice genes.** Click here for file

Additional file 16 **CNMS marker based comparative physical mapping showing colinearity between rice chromosome 1 and homeologous chromosomes of four other cereal species and *Arabidopsis thaliana*.** Click here for file

Additional file 17 **List of 18 rice genotypes used for studying the polymorphic potential of 60 GNMS markers and their comparison with 15 microsatellite markers designed from CDS.** Click here for file
