# Supplementary material for: Genic non-coding microsatellites in the rice genome: characterization, marker design and use in assessing genetic and evolutionary relationships among domesticated groups
Source: BMC Genomics. 2009 Mar 31;10:140. doi: 10.1186/1471-2164-10-140 (PMC2680414; doi:10.1186/1471-2164-10-140)
Supplement: Additional file 2 — Frequency and abundance of various microsatellite repeat-motif classes in the genic non-coding and coding sequences of the rice genome. [file 1471-2164-10-140-S2.doc]

**Additional file 2: Frequency and abundance of various microsatellite repeat-motif classes in the genic non-coding and coding sequences of rice genome. Trinucleotide GNMS repeat-motifs were most frequent in the 5’UTRs followed by promoters and minimum in the intronic sequences, whereas dinucleotide GNMS motifs were maximum in the introns and minimum in the 5’UTRs. Abundance of trinucleotide GNMS repeat-motifs in the CDS as compared with those of genic non-coding sequence components of rice genes was observed.**
