# Supplementary material for: Genic non-coding microsatellites in the rice genome: characterization, marker design and use in assessing genetic and evolutionary relationships among domesticated groups
Source: BMC Genomics. 2009 Mar 31;10:140. doi: 10.1186/1471-2164-10-140 (PMC2680414; doi:10.1186/1471-2164-10-140)
Supplement: Additional file 3 — Development of rice GNMS markers and their efficiency in detecting polymorphism. [file 1471-2164-10-140-S3.doc]

**Additional file 3 (A)**

**Additional file 3 (B)**

**Additional file 3 (C)**

**Additional file 3 (D)**

**Additional file 3 (E)**

**Additional file 3: Polymorphism survey using the GNMS markers designed from (GA)_13_ repeat-motif in the promoter sequence of β-galactosidase gene (A), (GAC)_7_ motif in the 5’UTR of Protein kinase gene (B), (AAAT)_5_ in the intron of NBS-LRR disease resistance gene (C), (GAA)_10_ in the CDS of Phospholipase gene (D) and (CATATA)_5_ in the 3’UTR of ATP-NAD kinase gene (E). Lanes: 1-IR64, 2-IR24, 3-Jaya, 4-Swarna, 5-Kalinga3, 6-Ratna, 7-Heera, 8-PusaBasmati1, 9-Kasturi, 10-Pusa1121, 11-CSR30, 12-TaraoriBasmati, 13-Basmati370, 14-Kalanamak, 15-Sonasal, 16-Bindli, 17-Taepai309, 18-Nipponbare and M-50 bp DNA ladder. Arrows indicate the polymorphic amplicons.**
