# Supplementary material for: Genic non-coding microsatellites in the rice genome: characterization, marker design and use in assessing genetic and evolutionary relationships among domesticated groups
Source: BMC Genomics. 2009 Mar 31;10:140. doi: 10.1186/1471-2164-10-140 (PMC2680414; doi:10.1186/1471-2164-10-140)
Supplement: Additional file 5 — GNMS marker based physical bin map of rice genome. [file 1471-2164-10-140-S5.doc]

**04-1**

**04-2**

**04-3**

**04-4**

**04-5**

**04-6**

**04-7**

**04-8**

**04-9**

**04-10**

**04-11**

**04-12**

**04-13**

**04-14**

**04-15**

**04-16**

**04-17**

**04-18**

**04-19**

**04-20**

**04-21**

**04-22**

**04-23**

**04-24**

**04-25**

**04-26**

**04-27**

**04-28**

**04-29**

**04-30**

**04-31**

**04-32**

**04-33**

**04-34**

**04-35**

**04-36**

**1424^i^, 1425^i^, 1426^p^, 1427^i^, 1428^f^, 1429^i^, 1430^f^, 1431^i^, 1432^p^, 1433^i^, 1434^i^, 1435^t^, 1436^p^, 1437^p^**

**1438^p,i^, 1439^i^, 1440^i^, 1441^i^, 1442^p^, 1443^p^, 1444^p^, 1445^i^, 1446^i^, 1447^i^, 1448^i^**

**1449^i^, 1450^i^, 1451^i^**

**1452^i^, 1453^f^, 1454^i^, 1455^i^**

**1456^i^, 1457^i^, 1458^i^**

**1459^i^, 1460^p^, 1461^i^, 1462^i^, 1463^i^**

**1464^p^, 1465^i^, 1466^i^, 1467^i^, 1468^i^**

**1469^i^, 1470^i^, 1471^i^, 1472^p^, 1473^i^, 1474^i^**

**1475^f^, 1476^i^**

**1477^i^**

**1478^i^, 1479^i^, 1480^p^, 1481^i^, 1482^i^, 1483^i^, 1484^i^**

**1485^i^, 1486^t^, 1487^i^, 1488^i^, 1489^i^**

**1490^i^, 1491^p^, 1492^f^, 1493^i^, 1494^f,p^, 1495^i^, 1496^p^**

**1497^i^, 1498^f^, 1499^i^, 1500^i^**

**1501^i^, 1502^i^**

**1503^i^, 1504^f^, 1505^i^, 1506^i^, 1507^i^, 1508^i^, 1509^i^, 1510^p^**

**1511^i^, 1512^i^, 1513^i^, 1514^i^**

**1515^p^, 1516^i^, 1517^i^, 1518^i^, 1519^f^**

**1520^i^, 1521^i^, 1522^f^, 1523^i^, 1524^p^, 1525^i^, 1526^i^, 1527^p^, 1528^i^, 1529^i^, 1530^p^, 1531^f^, 1532^f^, 1533^p^, 1534^f^, 1535^f^, 1536^i^, 1537^i^, 1538^i^, 1539^i^**

**1540^i^, 1541^i^, 1542^f^, 1543^i^, 1544^i^, 1545^i^, 1546^i^**

**1547^i^, 1548^p^, 1549^i^, 1550^f^, 1551^i^, 1552^p^, 1553^f^, 1554^i^, 1455^i^, 1456^p^, 1457^t^, 1458^i^, 1459^i^, 1460^f^**

**1561^i,p^, 1562^i^, 1563^i,p^, 1564^i^, 1565^f^, 1566^f^, 1567^t^, 1568^i^, 1569^f^, 1570^p^, 1571^p^**

**1572^p^, 1573^i^, 1574^p^, 1575^p^, 1576^i^, 1577^t^, 1578^f^, 1579^f^, 1580^i^, 1581^i^, 1582^i^, 1583^i^, 1584^f^**

**1585^p^, 1586^i^, 1587^f^, 1588^p^, 1589^i,f^, 1590^i^, 1591^i^, 1592^f^, 1593^i^, 1594^p^, 1595^i^, 1596^f^, 1597^i^, 1598^i^, 1599^f^, 1600^i^, 1601^i^**

**1602^i^, 1603^i^, 1604^i^, 1605^p^, 1607^i^, 1608^f^, 1609^i^, 1610^i^, 1611^p^, 1612^i^, 1613^p^, 1614^p^, 1615^i^, 1616^i^, 1617^i^, 1618^p^, 1619^i^, 1620^p^**

**1621^p^, 1622^f^, 1623^f^, 1624^i^, 1625^i^, 1626^p^, 1627_p_, 1628^i^, 1629^p^, 1630^i^, 1631^f^, 1632^p^, 1633^f^**

**1634^f^, 1635^f^, 1636^i^, 1637^i^, 1638^i^, 1639^i^, 1640^p^, 1641^i^**

**1642^i^, 1643^i^, 1644^p^,1645^i^, 1646^i^, 1647^p^, 1648^i^, 1649^i^**

**1650^i^, 1651^f^, 1652^p^, 1653^f^, 1654^f^, 1655^i^, 1656^p^, 1657^i^**

**1658^i^, 1659^f^, 1660^i^, 1661^t^, 1662^f^, 1663^i^, 1664^p^, 1665^i^, 1666^p^, 1667^f^, 1668^i^, 1669^i^, 1670^i^, 1671^i^**

**1672^i^, 1673^i^, 1674^f^, 1675^p^, 1676^i^, 1677^i^, 1678^p^, 1679^f^, 1680^i^, 1681^i^, 1682^i^, 1683^i^**

**1684^p^, 1685^f^, 1686^i^, 1687^p^, 1688^i^, 1689^i^, 1690^t^, 1691^f^, 1692^i^, 1693^p^, 1694^p,i^, 1695^f^, 1696^i^, 1697^i^, 1698^i^, 1699^f^**

**1700^f^, 1701^p^, 1702^i,t^, 1703^f,i^, 1704^f^, 1705^i^, 1706^i^, 1707^i^, 1708^p^, 1709^f^, 1710^f^, 1711^p^, 1712^p^, 1713^p^**

**1714^i^, 1715^i^, 1716^p^, 1717^p^, 1718^f^, 1719^i^, 1720^i^, 1721^i^, 1722^p^, 1723^i^, 1724^p^, 1725^f^, 1726^p^, 1727^f^, 1728^i^, 1729^p^,1730^i^, 1731^f^, 1732^i^, 1733^f^**

**1734^f^, 1735^i^, 1736^i^, 1736^i^**

**1737^i^, 1738^f^, 1739^f^**

**01-1**

**01-2**

**01-3**

**01-4**

**01-5**

**01-6**

**01-7**

**01-8**

**01-9**

**01-10**

**01-11**

**01-12**

**01-13**

**01-14**

**01-15**

**01-16**

**01-17**

**01-18**

**01-19**

**01-20**

**01-21**

**01-22**

**01-23**

**01-24**

**01-25**

**01-26**

**01-27**

**01-28**

**01-29**

**01-30**

**01-31**

**01-32**

**01-33**

**01-34**

**01-35**

**01-36**

**01-37**

**01-38**

**01-39**

**01-40**

**01-41**

**01-42**

**01-43**

**01-44**

**1^i^, 2 ^i^, 3 ^i^, 4 ^i^, 5^p^, 6 ^i^, 7^f, p^, 8^i^, 9^i,p^, 10^i^, 11^i^, 12^i^, 13^i^, 14^f^, 15^p^, 16^i^, 17^i^, 18^i^, 19^t^, 20^i^**

**21^i^, 22^p^, 23^i^, 24^i^, 25^i^, 26^p^, 27^p^, 28^i^**

**29^p^, 30^t^, 31^f^, 32^i^, 33^i^, 34^f^, 35^i^**

**36^p^, 37^f^, 38^i^, 39^f^, 40^f^, 41^p^, 42^i^, 43^t^, 44^p^, 45^i^, 46^p^, 47^i^**

**48^i,f^, 49^i^, 50^p^, 51^i,p^, 52^i^, 53^i,t^, 54^i^, 55^t^, 56^p^, 57^f^, 58^i^, 59^f^, 60^p^, 61^t^**

**62^i^, 63^p^, 64^i^, 65^i^, 66^f^, 67^i^, 68^t^, 69^p^, 70^i^, 71^i^, 72^f^**

**73^t^, 74^t^, 75^p^, 76^p,f^, 77^i^, 78^i^, 79^f^, 80^i^, 81^f^, 82^i^**

**83^p^, 84^t^, 85^p^, 86^i^, 87^i^, 88^p^, 89^i^, 90^i,f^, 91^p^, 92^i^, 93^f^, 94^p^, 95^i^, 96^f^, 97^i^, 98^i^, 99^f^, 100^f^, 101^t^, 102^f^, 103^i^, 104^i^, 105^f^, 106^i^, 107^f^, 108^i^, 109^i^, 110^f^, 111^f^**

**112^t^, 113^t^, 114^i^, 115^f,i^, 116^i^, 117^i^, 118^f,i^, 119^i^, 120^p^, 121^i^**

**122^i^, 123^f^, 124^f^, 125^i^, 126^f^, 127^p^, 128^t^, 129^f^, 130^i^**

**131^f^, 132^i^, 133^i^, 134^i^, 135^i^, 136^p^, 137^i^, 138^f^, 139^i^, 140^i^, 141^i^, 142^f,p^**

**143^i^, 144^i^, 145^p^, 146^i^, 147^t^, 148^i^, 149^t^, 150^i^, 151^i^, 152^i^, 153^i^**

**154^f^, 155^i^, 156^p^, 157^t^, 158^i^**

**159^i^, 160^i^, 161^i^, 161^i^, 162^f,p^, 163^f^, 164^i^, 165^p^, 166^i^, 167^i^, 168^i^, 169^i^, 170^p^, 171^f^, 172^i^**

**173^i^, 174^i^, 175^i^, 176^i^, 177^f^, 178^p^, 179^f^, 180^i, f^, 181^i^, 182^i^, 183^i^**

**184^f^, 185^f^, 186^p^, 187^p^, 188^i^, 189^i^**

**190^i^, 191^p^, 192^p^, 193^i^**

**194^t^, 195^f^, 196^i^**

**197^p^, 198^p^, 199^f,i^, 200^f^, 201^i^, 202^p^, 203^i^**

**204^t^, 205^f^, 206^p,i^, 207^p^, 208^f^, 209^i^, 210^i^**

**211^f^, 212^i^, 213^p^, 214^i^, 215^p,i^, 216^i^, 217^i^, 218^p^, 219^i^**

**220^i^, 221^p^, 222^i^, 223^i^, 224^f^, 225^i,f^, 226^i^, 227^f^, 228^i^, 229^i^, 230^p^**

**231^t^, 232^i,p^, 233^p^, 234^i^, 235^i^, 236^i^, 237^i^, 238^f^, 239^i^, 240^i^**

**241^t^, 242^f^, 243^i^, 244^f^, 245^i^, 246^i^, 247^p^, 248^i^, 249^i^, 250^i^**

**251^i^, 252^i^, 253^i^, 254^i^, 255^f^, 256^i^, 257^p^, 258^f^, 259^i^, 260^f^, 261^i^**

**262^f^, 263^f^, 264^p^, 265^i^, 266^f^, 267^f^, 268^i^, 269^i^**

**270^i^, 271^i^, 272^i^**

**273^i^, 274^i^, 275^i^, 276^f^, 277^i^, 278^f^, 279^i^, 280^p^, 281^p,f^, 282^t^, 283^p^, 284^f^**

**285^f^, 286^t^, 287^i^, 288^f^, 289^i^, 290^i^, 291^i^, 292^f^, 293^i^, 294^i^, 295^p^**

**296^p^, 297^t^, 298^t^, 299^i^, 300^i^, 301^t^, 302^p^, 303^i^, 304^i, p^, 305^i^, 306^p^**

**307^f^, 308^t^, 309^p^, 310^p^, 311^i^, 312^f^, 313^p^, 314^f^, 315^i^, 316^p^, 317^p^, 318^f^, 319^i^**

**320^p^, 321^i^, 322^f^, 323^i^, 324^f^, 325^i^, 326^i^, 327^i^, 328^p^, 329^p^, 330^i^, 331^t^, 332^f^, 333^f^, 334^p^**

**335^p^, 336^f^, 337^i^, 338^i^, 339^i^, 340^p^, 341^t^, 342^f^, 343^i^, 344^i^**

**345^i^, 346^f^, 347^f^, 348^i^, 349^f^, 350^i^, 351^i^, 352^i^, 353^i^, 354^f,i^, 355^i^, 356^i^, 357^i^, 358^p^**

**359^p^, 360^i^, 361^p^, 362^i^, 363^i^, 364^f^, 365^i^, 366^i^, 367^i^, 368^i^, 369^f^, 370^i^, 371^i^**

**372^f^, 373^f^, 374^i,f^, 375^p^, 376^f^, 377^t^, 378^i^, 379^i^, 380^f^, 381^t^, 382^i,p^, 383^i^, 384^i^**

**385^i^, 386^f,i^, 387^f^, 388^i^, 389^f^, 390^f^, 391^i^, 392^i^, 393^f^, 394^i^, 395^i^, 396^i^, 397^i^, 398^i^, 399^f^**

**400^i^, 401^p^, 402^f^, 403^i^, 404^i^, 405^t^, 406^p^, 407^p^, 408^p^, 409^i^, 410^f^, 411^i^**

**412^f^, 413^i^, 414^i, p^, 415^p^, 416^t^, 417^i,f^, 418^i^, 419^f^, 420^f^, 421^f^, 422^i^, 423^i^, 424^i^, 425^p^, 426^t^**

**427^i^, 428^i^, 429^f^, 430^i^, 431^i^, 432^f^, 433^f^, 434^i^, 435^i^, 436^p^, 437^i^, 438^i^**

**439^i^, 440^p^, 441^p^, 442^p^, 443^p^, 444^i^, 445^p^, 446^f^, 447^i^, 448^i^, 449^f^, 450^i^, 451^p^, 452^f^, 453^i^, 454^f^, 455^i^**

**456^f^, 457^i^, 458^f^, 459^f^, 460^f^, 461^p^, 462^i^**

**463^i^, 464^f^, 465^i^, 466^i^, 467^i, t^, 468^i^, 469^i^, 470^p^, 471^p^, 472^i^**

**473^i^, 474^i^, 475^f^, 476^i^, 477^t^, 478^i^, 479^i^, 480^i^, 481^i^, 482^i^, 483^i^, 484^p^, 485^i^, 486^f^, 487^t,i^**

**1**

**2**

**3**

**4**

**5**

**6**

**02-1**

**02-2**

**02-3**

**02-4**

**02-5**

**02-6**

**02-7**

**02-8**

**02-9**

**02-10**

**02-11**

**02-12**

**02-13**

**02-14**

**02-15**

**02-16**

**02-17**

**02-18**

**02-19**

**02-20**

**02-21**

**02-22**

**02-23**

**02-24**

**02-25**

**02-26**

**02-27**

**02-28**

**02-29**

**02-30**

**02-31**

**02-32**

**02-33**

**02-34**

**02-35**

**02-36**

**488^i^, 489^p^, 490^p^, 491^i,f^, 492^i^, 493^p^, 494^i^, 495^f^, 496^i^, 497^f^, 498^t^, 499^f^, 500^i^, 501^i^, 502^i^, 503^i^, 504^i^, 505^p^, 506^i^, 507^i^, 508^i^, 509^f^, 510^t^, 511^p^**

**512^p^, 513^f^, 514^i^, 515^p^, 516^f^, 517^i^, 518^p^, 519^i^, 520^f^, 521^i^, 522^f^, 523^f^, 524^p^, 525^p^, 526^i^, 527^i^, 528^i^, 529^p^, 530^t^, 531^f^, 532^p^, 533^f^, 534^p^**

**535^p,i^, 536^p,i^, 537^i^, 538^p^, 539^i^, 540^p^, 541^f,i^, 542^p^, 543^i^, 544^p^**

**545^i^, 546^i^, 547^f^, 548^f^, 549^i^, 550^i^, 551^p^, 552^f^, 553^f^, 554^i^, 555^i^, 556^i^, 557^i^, 558^f^, 559^i^**

**560^f^, 561^p^, 562^i^, 563^i^, 564^i^, 565^i^, 566^i^, 567^i^, 568^i^, 569^i^, 570^p^, 571^i^, 572^i^, 573^p^, 574^f^, 575^f^, 576^i^, 577^i^, 578^i^, 579^p^, 580^i^, 581^f^, 582^i^**

**583^p^, 584^f^, 585^f^, 586^p^, 587^i^, 588^p^, 589^i^, 590^t,f^, 591^p^, 592^i^, 593^p^, 594^i^, 595^i^, 596^f^, 597^i^, 598^f^, 599^f^, 600^f^, 601^i^, 602^f^, 603^i^, 604^p^, 605^p^**

**606^i^, 607^i^, 608^i^, 609^p^, 610^p^, 611^i^, 612^p^**

**613^i^, 614^t^, 615^i^, 616^f^, 617^f^, 618^f^, 619^f^, 620^i^, 621^i^, 622^i^, 623^i^, 624^i^, 625^i^**

**626^i^, 627^p^, 628^p^, 629^i^, 630^i^, 631^i^, 632^p, i^, 633^p^, 634^f^, 635^i^, 636^p^, 637^p^**

**638^f^, 639^f^, 640^i^, 641^f^, 642^p^, 643^i^, 644^i^, 645^i^, 646^f^**

**647^f^, 648^t,i^, 649^f^, 650^f^, 651^i^, 652^i^, 653^i^, 654^f^, 655^i^, 656^i^, 657^i^, 658^f^, 659^p^**

**660^i^, 661^i^, 662^i^, 663^i^, 664^i^, 665^i^, 666^i^, 667^i^, 668^i^**

**669^i^, 670^i^, 671^p^, 672^i^, 673^f^, 674^i^**

**675^p^, 676^i^, 677^f^, 678^i^, 679^i^, 680^i^**

**681^p^, 682^i^, 683^i^**

**684^i^, 685^p^, 686^i^, 687^i^, 688^f^, 689^i^, 690^i^, 691^i^**

**692^f^, 693^p^, 694^i^, 695^i^, 696^f^, 697^i^, 698^t^, 699^t^, 700^i^, 701^p^**

**702^p^, 703^i^, 704^f^, 705^f^, 706^i^**

**707^f^, 708^i^, 709^t^, 710^i^, 711^i^, 712^t^, 713^i^, 714^i^, 715^i^, 716^p^, 717^i^**

**718^i^, 719^i^, 720^t^, 721^i^, 722^i^, 723^f^, 724^f^, 725^i^, 726^i^, 727^i^, 728^i^**

**729^p^, 730^i^, 731^i^, 732^i^, 733^i^, 734^i^, 735^i^, 736^p^, 737^f,i^, 738^i^**

**739^f^, 740^i^, 741^i^, 742^f^, 743^p^, 744^i^, 745^p^, 746^i^, 747^i^, 748^i^, 749^i^**

**750^i^, 751^p^, 752^p^, 753^i^, 754^i^, 755^i^, 756^i^, 757^i^, 758^i^, 759^f^,**

**760^f^, 761^f^**

**762^i^, 763^f^, 764^p^, 765^i^, 766^p,i^, 767^i^, 768^p^, 769^i,f^, 770^i^, 771^i,f^, 772^f^, 773^i^, 774^i^, 775^i^, 776^t^**

**777^i^, 778^p^, 779^i^, 780^i^, 781^i^, 782^f^, 783^f^, 784^i^, 785^i^, 786^f^, 787^i^**

**788^f^, 789^p^, 790^p^, 791^i,t^, 792^i^, 793^i^, 794^i^, 795^t^, 796^p,i^, 797^i^, 798^p^, 799^f^, 800^f^, 801^p^**

**802^f^, 803^i^, 804^t^, 805^i^, 806^i^, 807^f^, 808^i^, 809^p^, 810^p^, 811^p^**

**812^i^, 813^i^, 814^i^, 815^i^, 816^f^, 817^f^, 818^i^, 819^p^, 820^i^, 821^i^**

**822^p^, 823^f^, 824^i^, 825^p^, 826^f^, 827^i^, 828^i^, 829^i^, 830^f^, 831^f^, 832^i^, 833^p^, 834^i^, 835^i^**

**836^f^, 837^f^, 838^p^, 839^i^, 840^f^, 841^i^, 842^i^, 843^i^, 844^i^, 845^p^, 846^p^, 847^p^, 848^f^**

**849^i^, 850^i^, 851^p^, 852^i^, 853^i^, 854^p^, 855^f^, 856^i^, 857^p^, 858^p^, 859^i^, 860^f^, 861^p^, 862^p^, 863^i^, 864^p^, 865^t^**

**866^i^, 867^f,i^, 868^f^, 869^i^, 870^p^, 871^f^, 872^p^, 873^p^**

**874^i^, 875^i^, 876^i,p^, 877^f^, 878^i^, 879^f^, 880^i^, 881^f,i^, 882^i^, 883^i^, 884^i^, 885^i^**

**886^p^, 887^f^, 888^f^, 889^f^, 890^i^, 891^p^, 892^f^, 893^f^, 894^i^, 895^t^, 896^p^, 897^f^, 898^p^**

**899^i^, 900^t^, 901^i^, 902^i^, 903^i^, 904^i^, 905^i^, 906^i^, 907^f^, 908^i,p^, 909^i,p^, 910^i^, 911^i,p^, 912^i^, 913^p^, 914^i^, 915^i^, 916^t^, 917^p^, 918^i^**

**919^i^, 920^t^, 921^i^, 922^f^, 923^i,f^ , 924^p^, 925^f^, 926^f^, 927^f^, 928^p^, 929^i^, 930^i^, 931^i^, 932^i^, 933^i^, 934^i^, 935^t^, 936^t^**

**937^f^, 938^i^, 939^f^, 940^t^, 941^i^, 942^i^, 943^i^, 944^p^, 945^i^, 946^i^, 947^i,f^, 948^f^, 949^i^, 950^i^, 951^t^, 952^t^, 953^f^, 954^i^, 955^f,i^, 956^i,f^, 957^i^, 958^f^**

**959^i^, 960^i,p^, 961^i^, 962^f^, 963^i^, 964^f^, 965^f^, 966^i^, 967^p^, 968^p^, 969^i^, 970^f^, 971^f^, 972^p^, 973^f^**

**974^i^, 975^f^, 976^p^, 977^i^, 978^i^, 979^t,i^, 979^i^, 980^i^, 981^i^, 982^f^, 983^p^, 984^t^, 985^p^, 986^f^, 987^i^**

**988^p^, 989^f^, 990^i^, 991^i^, 992^f^, 993^i^, 994^t^, 995^f^, 996^t^, 997^f^, 998^i^, 999^i^, 1000^i^, 1001^f^, 1002^i^, 1003^f^, 1004^t^, 1005^i^, 1006^p^, 1007^t^, 1008^p^, 1009^f^**

**1010^i^, 1011^p^, 1012^i^, 1013^p^, 1014^i^, 1015^i^, 1016^i^, 1017^f^, 1018^i^, 1019^f^, 1020^p^, 1021^f^, 1022^i^**

**1023^f^, 1024^p^, 1025^f^, 1026^i^, 1027^i^, 1028^i^, 1029^f^, 1030^i^, 1031^p^, 1032^i^, 1033^i^, 1034^i^, 1035^i^, 1036^f^, 1037^i^**

**1038^f,t^, 1039^i^, 1040^p^, 1041^i^, 1042^i^, 1043^f^, 1044^f^, 1045^i,p^, 1046^f^, 1047^p^**

**1048^f^, 1049^i^, 1050^p,i^, 1051^p^, 1052^i^, 1053^i^, 1054^f^, 1055^i^, 1056^t^, 1057^i^, 1058^f^, 1059^f^, 1060^p^, 1061^i^, 1062^i^, 1063^p^**

**1064^f^, 1065^f^, 1066^i^, 1067^i^, 1068^p^, 1069^p^, 1070^f^, 1071^p^, 1072^i^, 1073^p^**

**1074^f^, 1075^f^, 1076^f^, 1077^p^, 1078^f,p^, 1079^p^, 1080^i^, 1081^i^, 1082^p^, 1083^f^, 1084^i^, 1085^f^, 1086^i^, 1087^p^, 1088^i^, 1089^i^, 1090^i^**

**1091^i^, 1092^p^, 1093^i^, 1094^i^, 1095^i^, 1096^p^, 1097^p^, 1098^f^, 1099^i^, 1100^f^, 1101^p^, 1102^f,p^, 1103^i^, 1104^p^, 1105^i^, 1106^f^**

**1107^p^, 1108^p^, 1109^f^, 1110^f^, 1111^f^, 1112^p^, 1113^i^, 1114^t^, 1115^p^, 1116^i^**

**1117^f^, 1118^p^, 1119^f^, 1120^i^, 1121^i^, 1122^i^, 1123^t^, 1124^i^, 1125^i^, 1126^p^, 1127^p^**

**1128^f^, 1129^f^, 1130^i^, 1131^f^, 1132^f^, 1133^p^, 1134^f^, 1135^i^, 1136^i^, 1137^f^, 1138^f^, 1139^f^, 1140^i^, 1141^i^, 1142^f^, 1143^f^, 1144^i^, 1145^f^, 1146^f^**

**1147^i^, 1148^i^, 1149^t^, 1150^i^, 1151^i,p^, 1152^t^, 1153^p^, 1154^i^, 1155^p^, 1156^f^, 1157^f^, 1158^i^**

**1159^i^, 1160^p^, 1161^i^, 1162^i^, 1163^f^, 1164^p^, 1165^p^, 1166^p^, 1167^i^, 1168^i^, 1169^f^, 1170^f^, 1171^i^, 1172^i^, 1173^i^, 1174^i^, 1175^i^, 1176^i^, 1177^i^**

**1178^i^, 1179^p^, 1180^f^, 1181^i^, 1182^p^, 1183^f^, 1184^i^, 1185^i^, 1186^f^, 1187^f^, 1188^i^, 1189^i^, 1190^i^, 1191^i^, 1192^f^**

**1193^i^, 1194^f^, 1195^i^, 1196^i^, 1197^f^, 1198^i^, 1199^i^, 1200^f^, 1201^t^, 1202^i^, 1203^i^**

**1204^p^, 1205^i^, 1206^f^, 1207^i^, 1208^f^, 1209^f^, 1210^i^**

**1211^p^, 1212^i,p^, 1213^f^, 1214^i^**

**1215^i^, 1216^p^, 1217^i^, 1218^i^, 1219^i^, 1220^i^**

**1221^i^, 1222^i^, 1223^i^, 1224^f^, 1225^i^, 1226^i^, 1227^t^, 1228^i^**

**1229^i^, 1230^f^, 1231^i^, 1232^i^, 1233^f^, 1234^f^, 1235^f^, 1236^i^, 1237^p^, 1238^i^, 1239^i^**

**1240^i^, 1241^i^, 1242^p^, 1243^p^, 1244^i^, 1245^i^, 1246^i^, 1247^i^, 1248^i^, 1249^i^, 1250^p^, 1251^i^, 1252^f^, 1253^i^**

**1254^t^, 1255^i^, 1256^i^, 1257^i^, 1258^f^, 1259^i^, 1260^f^, 1261^p^, 1262^i^, 1263^i^, 1264^f^**

**1265^i^, 1266^i^, 1267^p^, 1268^i^, 1269^p^, 1270^i^, 1271^i,t^, 1272^f^, 1273^i^, 1274^f^, 1275^i^, 1276^p^, 1277^f^, 1278^p^, 1279^i^, 1280^t^, 1281^i^**

**1282^p,i^, 1283^i^, 1284^p^, 1285^p^, 1286^f^, 1287^i^, 1288^i^, 1289^i^, 1290^i^, 1291^f^, 1292^f^, 1293^i^**

**1294^i^, 1295^f^, 1296^f^, 1297^f^, 1298^p^, 1299^f^, 1300^i^, 1301^f^, 1302^i^, 1303^i^, 1304^p^, 1305^f^, 1306^i^**

**1307^p^, 1308^i^, 1309^f^, 1310^p^, 1311^f^, 1312^p^, 1313^i^, 1314^i^, 1315^i^, 1316^i^, 1317^i^, 1318^p^, 1319^i^**

**1320^f^, 1321^f^, 1322^i^, 1323^f^, 1324^f^, 1325^i^, 1326^f^, 1327^f^, 1328^f^, 1329^i^, 1330^i^, 1331^i^, 1332^f^, 1333^i^, 1334^i^, 1335^f^, 1336^p^, 1337^p^, 1338^i^, 1339^i^, 1340^i^, 1341^p^**

**1342^p^, 1343^i^, 1344^i^, 1345^p^, 1346^i^, 1347^t^, 1348^t^, 1349^t^, 1350^i^, 1351^i^, 1352^i^, 1353^p,i^, 1354^i^, 1355^f^, 1356^p^, 1357^i^**

**1358^i^, 1359^f^, 1360^p^, 1361^i^, 1362^f^, 1363^i^, 1364^t^, 1365^i^, 1366^f^, 1367^f^, 1368^i^, 1369^p^**

**1370^p^, 1371^i^, 1372^i^, 1373^f^, 1374^f^, 1375^i^, 1376^p^, 1377^f^, 1378^f^, 1379^f^, 1380^i^, 1381^i^, 1382^i^, 1383^i^**

**1384^i^, 1385^i^, 1386^i^, 1387^i^, 1388^f^, 1389^f^, 1390^i^, 1391^i^, 1392^f^, 1393^i^, 1394^f^, 1395^i^, 1396^p^**

**1397^f^, 1398^i^, 1399^i^, 1400^p^, 1401^i^, 1402^i^, 1403^f^, 1404^i^, 1405^i^, 1406^i,f^, 1407^p^, 1408^f^, 1409^i^, 1410^i^, 1411^i^, 1412^f^, 1413^f^**

**1414^i^, 1415^i^, 1416^t^**

**1417^p^, 1418^f^, 1419^f^, 1420^i^, 1421^i^, 1422^f,i^, 1423^i^**

**03-1**

**03-2**

**03-3**

**03-4**

**03-5**

**03-6**

**03-7**

**03-8**

**03-9**

**03-10**

**03-11**

**03-12**

**03-13**

**03-14**

**03-15**

**03-16**

**03-17**

**03-18**

**03-19**

**03-20**

**03-21**

**03-22**

**03-23**

**03-24**

**03-25**

**03-26**

**03-27**

**03-28**

**03-29**

**03-30**

**03-31**

**03-32**

**03-33**

**03-34**

**03-35**

**03-36**

**03-37**

**05-1**

**05-2**

**05-3**

**05-4**

**05-5**

**05-6**

**05-7**

**05-8**

**05-9**

**05-10**

**05-11**

**05-12**

**05-13**

**05-14**

**05-15**

**05-16**

**05-17**

**05-18**

**05-19**

**05-20**

**05-21**

**05-22**

**05-23**

**05-24**

**05-25**

**05-26**

**05-27**

**05-28**

**05-29**

**05-30**

**1740^i^, 1741^f^, 1742^i^, 1743^i^, 1744^i^, 1745^f^, 1746^f^, 1747^i^, 1748^i^, 1749^p^, 1750^i^, 1751^i^, 1752^p^, 1753^i^, 1754^t^ , 1755^i^, 1756^p^, 1757^f^, 1758^i^, 1759^f^, 1760^i^, 1761^i^, 1762^f^, 1763^i^**

**1764^i^, 1765^t^, 1766^i^, 1767^t^, 1768^p^, 1769^i^, 1770^p^, 1771^i^, 1772^i^**

**1773^f^, 1774^i^, 1775^p,i^, 1776^i^, 1777^i^, 1778^p^, 1779^i^, 1780^f^, 1781^i^, 1782^i^, 1783^i^, 1784^i^, 1785^p^, 1786^i,f^, 1787^p^, 1788^i^, 1789^p^, 1790^f^, 1791^i^, 1792^i^**

**1793^i^, 1794^i^, 1795^i^, 1796^p^, 1797^i^, 1798^i^, 1799^i^, 1800^i^, 1801^i^**

**1802^i^, 1803^f^, 1804^i^, 1805^p^, 1806^f^, 1807^f^, 1808^i^, 1809^i^, 1810^i^, 1811^f,i^**

**1812^i^, 1813^p^, 1814^i^, 1815^i^, 1816^p^, 1817^f^, 8118^p^, 1819^i^**

**1820^f^, 1821^p^, 1822^i^, 1823^t,p^, 1824^i^, 1825^t^, 1862^i^, 8172^p^, 1828^f^, 1829^p^, 1830^i^, 8131^i,t^**

**1832^i^, 1833^f^, 1834^i^, 1835^f^, 1836^f^, 1837^t^, 1838^i^, 1839^i,f^**

**1840^f^, 1841^i^, 1842^i^, 1843^i^, 1844^p^, 1845^p^, 1846^i^, 1847^t^**

**1848^i^, 1849^i^, 1850^i^, 1851^p^, 1852^f^, 1853^f^, 1854^i^, 1855^i^, 1856^i^**

**1857^i^, 1858^i^, 8159^i^, 1860^i^, 1861^i^**

**1862^i^, 1863^i^, 1864^i^, 1865^i^, 1866^i^, 1867^i^**

**1868^i^, 1869^p^**

**1870^p^, 1871^p^, 1872^f^, 1873^i^, 1874^i,p^, 1875^f^, 1876^f^, 1877^p^, 1878^p^, 1879^t^**

**1880^i^, 1881^i^, 1882^i^, 1883^p^, 1884^i^, 1885^i,f^, 1886^i^, 1887^i^**

**1888^i^, 1889^f^, 1890^i^, 1891^i^, 1892^i^**

**1893^i^, 1894^p^, 1895^i^, 1896^f^, 1897^i^, 1898^i^, 1899^i^, 1900^i^**

**1901^i^, 1902^i^, 1903^i^, 1904^i^, 1905^i^, 1906^f^, 1907^f^, 1908^i^, 1909^f^, 1908^i^, 1909^f^, 1910^i^, 1911^i^**

**1912^p^, 1913^i^, 1914^p^, 1915^t^, 1916^f^, 1917^f^, 1918^p^, 1919^f^, 1920^p^**

**1921^i^, 1922^i^, 1923^i^, 1924^i^, 1925^f^, 1926^p^, 1927^f^, 1928^i^, 1929^f^, 1930^f^, 1931^t^, 1932^i,f^**

**1933^f^, 1934^p^, 1935^i^, 1936^p^, 1937^i^, 1938^i^, 1939^i^, 1940^i^, 1941^f^, 1942^f^, 1943^p^, 1944^p^, 1945^i^, 1946^i^, 1947^i^**

**1948^i^, 1949^i^, 1950^p^, 1951^i^, 1952^f^, 1953^i^, 1954^f^, 1955^i^, 1956^t^, 1957^i^**

**1958^i^, 1959^i^, 1960^p^, 1961^p^, 1962^p^, 1963^t^, 1964^f^, 1965^f^, 1966^f^, 1967^f,i^, 1968^p^, 1969^p^, 1970^i^**

**1971^p^, 1972^i^, 1973^i^, 1974^p^, 1975^p^, 1976^f^, 1977^f,i^, 1978^p^, 1979^f^, 1980^p^, 1981^i^, 1982^f^, 1983^f^, 1984^f^, 1985^f^**

**1986^i,p^, 1987^i^, 1988^p^, 1989^i^, 1990^i^, 1991^i^, 1992^i^, 1993^p^, 1994^i^, 1995^p^, 1996^i^**

**1997^f^, 1998^f^, 1999^p^, 2000^f^, 2001^i,p^, 2002^i^, 2003^i^, 2004^i^, 2005^i^, 2006^i^, 2007^i^, 2008^f^, 2009^i^, 2010^i^**

**2011^t^, 2012^i^, 2013^f^, 2014^i^, 2015^p^, 2016^f^, 2017^i^, 2018^i^, 2019^i^**

**2020^i^, 2021^f^, 2022^p^, 2023^t^, 2024^f^, 2025^t^, 2026^f^, 2027^i^, 2028^f^, 2029^f^, 2030^p^, 2031^p^, 2032^f^, 2033^f^, 2034^i^, 2035^i^, 2036^i^, 2037^f^, 2038^t^**

**2039^p^, 2040^p^, 2041^t^, 2042^i^, 2043^i^, 2044^f^, 2045^p^, 2046^f^, 2047^p^, 2048^f^, 2049^i^, 2050^t^, 2051^f^, 2052^f^, 2053^i^, 2054^i^, 2055^i^, 2056^p^**

**2057^t^, 2058^f^, 2059^i^, 2060^p^, 2061^i^, 2062^i^, 2063^i^, 2064^i^, 2065^f^, 2066^i^, 2067^p^, 2068^i^, 2069^p^, 2070^i^, 2071^i^, 2072^p,t^, 2073^f^, 2074^t^, 2075^i^, 2076^f^, 2077^t^, 2078^i^**

**06-1**

**06-2**

**06-3**

**06-4**

**06-5**

**06-6**

**06-7**

**06-8**

**06-9**

**06-10**

**06-11**

**06-12**

**06-13**

**06-14**

**06-15**

**06-16**

**06-17**

**06-18**

**06-19**

**06-20**

**06-21**

**06-22**

**06-23**

**06-24**

**06-25**

**06-26**

**06-27**

**06-28**

**06-29**

**06-30**

**06-31**

**06-32**

**2079^i^, 2080^i^, 2081^f^, 2082^i^, 2083^i,f^, 2084^f^, 2085^p,t^, 2086^i^, 2087^p^, 2088^i^, 2089^i^, 2090^p^, 2091^p^, 2092^f^, 2093^i^, 2094^f^**

**2095^t^, 2096^i^, 2097^t^, 2098^i^, 2099^p^, 2100^p^, 2101^p^, 2102^i^, 2103^t^, 2104^p^, 2105^p^, 2106^p^, 2107^f^, 2108^i^, 2109^f,i^, 2110^p^, 2111^i^, 2112^i^**

**2113^t^, 2114^i^, 2115^t^, 2116^i^, 2117^p,i^, 2118^f^, 2119^f^, 2120^i^, 2121^i^, 2122^i^, 2123^i^, 2124^f^, 2125^p^, 2126^p^, 2127^i,f^, 2128^p^, 2129^i^**

**2130^i^, 2131^p^, 2132^i^, 2133^i^, 2134^i^, 2135^f^, 2136^p^, 2137^f^, 2138^f^, 2139^i^, 2140^i^, 2141^i^, 2142^f^, 2143^i^**

**2144^p^, 2145^i^, 2146^f^, 2147^p,i^, 2148^i^, 2149^f^, 2150^i,f^, 2151^i^, 2152^i^, 2152^i^, 2153^p^, 2154^f^, 2155^f^, 2156^f^, 2157^p^, 2158^i^**

**2159^i^, 2160^i^, 2161^i^, 2162^p^, 2163^p^, 2164^f^, 2165^i^, 2166^i^**

**2167^i^, 2168^f^, 2169^p^, 2170^i^, 2171^i^, 2172^i^, 2173^f^, 2174^p^, 2175^i^, 2176^p^, 2177^p^**

**2178^p^, 2179^p^, 2180^i^, 2181^i^, 2182^p^, 2183^i^**

**2184^i^, 2185^i^, 2186^i^, 2187^f^, 2188^f^, 2189^p^**

**2190^i^, 2191^t^, 2192^i^, 2193^p^, 2194^i^**

**2195^i^, 2196^p^**

**2197^f^, 2198^i^, 2199^f^, 2200^i^, 2201^i^, 2202^i^, 2203^f^, 2204^i^, 2205^i^**

**2206^p^, 2207^i^, 2208^p^, 2209^i^, 2210^f^, 2211^f^, 2212^f^, 2213^i^, 2214^p^, 2215^f^**

**2216^i^, 2217^p^, 2218^i^, 2219^i^, 2220^p^**

**2221^i^, 2222^t^, 2223^f^, 2224^i^**

**2225^i^, 2226^f^, 2227^i^**

**2228^i^, 2229^i^, 2230^p^, 2231^i^, 2232^p^, 2233^i^, 2234^i^**

**2235^f^, 2236^p^**

**2237^i^, 2238^f^, 2239^i^, 2240^i^, 2241^i^, 2242^i^, 2243^i^, 2244^i^, 2245^f^, 2246^f^, 2247^p^**

**2248^t^, 2249^i^, 2250^i^**

**2251^i^, 2252^f^, 2253^i^, 2254^f^, 2255^i^, 2256^i^, 2257^i^, 2258^f^, 2259^i,p^**

**2260^i^, 2261^i^, 2262^i^, 2263^i^, 2264^p^, 2265^i^, 2266^i^**

**2267^i^, 2268^p^, 2269^f^, 2270^t^, 2271^i^**

**2272^p^, 2273^t^, 2274^i^, 2275^i^, 2276^p^, 2277^p^, 2278^p^, 2279^i^, 2280^p^, 2281^i^, 2282^t^, 2283^i^, 2284^p^, 2285^i^, 2286^t^, 2287^p^**

**2288^i^, 2289^f^, 2290^i^, 2291^p^, 2292^p^, 2293^f^, 2294^i^, 2295^f,i^, 2296^p^, 2297^i^, 2298^f,t^, 2299^i^**

**2300^i^, 2301^i^, 2302^i^, 2303^p^, 2304^i^, 2305^f^, 2306^t^, 2307^f^**

**2308^f^, 2309^i^, 2310^p^, 2311^f^, 2312^f^, 2313^i^, 2314^i^, 2315^p^, 2316^f^, 2317^f^, 2318^i^**

**2319^i^, 2320^i^, 2321^i^, 2322^f^, 2323^i^, 2324^i^, 2325^i^, 2326^i^, 2327^p^, 2328^i^, 2329^t^, 2330^f^, 2331^p^, 2332^i^**

**2333^i^, 2334^t^, 2335^t,f^, 2336^f^, 2337^p^, 2338^f^, 2339^f^, 2340^i^, 2341^p^**

**2342^t^, 2343^f^, 2344^t,i^, 2345^f^, 2346^i^, 2347^i^, 2348^p^, 2349^i^, 2350^i^, 2351^p^, 2351^p^, 2352^p^, 2353^i^, 2354^i^, 2355^i^, 2356^i^, 2357^p^, 2358^p^, 2359^f^, 2360^i^, 2361^p^**

**2362^t,p^, 2363^i^**

**2364^i^, 2365^f^, 2366^t^, 2367^i^**

#### Additional file 5 (Continued)

**7**

**8**

**9**

**100**

**120**

**12-1**

**12-2**

**12-3**

**12-4**

**12-5**

**12-6**

**12-7**

**12-8**

**12-9**

**12-10**

**12-11**

**12-12**

**12-13**

**12-14**

**12-15**

**12-16**

**12-17**

**12-18**

**12-19**

**12-20**

**12-21**

**12-22**

**12-23**

**12-24**

**12-25**

**12-26**

**12-27**

**12-28**

**3612^i^, 3613^p^, 3614^p^, 3615^i^, 3616^i^, 3617^f^, 3618^i^, 3619^i^, 3620^p^, 3621^p^, 3622^f^, 3623^f^, 3624^p^, 3652^i^, 3626^i^, 3627^i^, 3628^i^**

**3629^p^, 3630^i^, 3631^i^, 3632^i^, 3633^i^, 3634^i^, 3635^i^, 3636^i^, 3637^f^, 3638^i^, 3639^p^, 3640^t^, 3641^i^, 3642^p^**

**3643^f^, 3644^f^, 3645^i^, 3646^f^, 3647^i^, 3648^p^, 3649^i^**

**3650^i^, 3651^t^, 3652^i^, 3653^f^, 3654^i^, 3655^i^, 3656^f^, 3657^f^, 3658^i^, 3659^i^, 3660^f^, 3661^i^, 3662^i^, 3663^f^, 3664^i^, 3665^t^, 3666^p^, 3667^i^, 3668^i^, 3669^i^, 3670^i^, 3671^i^, 3672^f^, 3673^f^, 3674^i^**

**3675^f^, 3676^i^, 3677^i^, 3678^p^, 3679^p^, 3680^f^, 3681^i^, 3682^i^, 3683^f^, 3684^i^**

**3685^f^, 3686^f^, 3687^i^, 3688^i^, 3689^i^, 3690^i^, 3691^i^, 3692^f^, 3693^i^**

**3694^i^, 3695^p^, 3696^i^, 3697^i^, 3698^i^**

**3699^p^, 3700^p^, 3701^i^, 3702^p^, 3703^i^, 3704^i^, 3705^i^, 3706^i^, 3707^i^, 3708^i^, 3709^t^, 3710^i^, 3711^i^**

**3712^f^, 3713^i^, 3714^i^**

**3715^i^, 3716^i^, 3717^i^, 3718^p^, 3719^i^, 3720^i^, 3721^i^**

**3722^i^, 3723^i^, 3724^f^, 3725^p^, 3726^f^, 3727^i^, 3728^f^, 3729^t^, 3730^i^**

**3731^p^, 3732^t^, 3733^i^**

**3734^i^, 3735^f^, 3736^i^, 3737^i^**

**3738^i^, 3739^p^**

**3740^i^, 3741^i^, 3742^p^, 3743^p^, 3744^i^**

**3745^i^, 3746^i^, 3746^i^, 3747^i^, 3748^f^, 3749^i^**

**3750^f^, 3751^i^**

**3752^i^, 3753^i^, 3754^i^**

**3755^f^, 3756^i^, 3757^i^, 3758^f^, 3759^i^, 3760^i^, 3761^i^**

**3762^i^, 3763^i^, 3764^i^, 3765^i^, 3766^i^, 3767^i^, 3768^f^, 3769^f^, 3770^i^, 3771^p^, 3772^f^, 3773^i^**

**3774^t^, 3775^i^, 3776^i^, 3777^p^**

**3778^i^, 3779^f^, 3780^f^, 3781^i^, 3782^f^, 3783^i^, 3784^t^, 3785^t^**

**3786^i^, 3787^i^, 3788^p^, 3789^f^, 3790^t^, 3791^t^, 3792^i^, 3793^p^, 3794^f^, 3795^f^, 3796^i^, 3797^f^**

**3798^f^, 3799^p^, 3800^i^, 3801^p^, 3802^f^, 3803^i^, 3804^i^, 3805^p^, 3806^i^, 3807^f^, 3808^f^, 3809^p^, 3810^i^, 3811^f^, 3812^t^, 3813^i^, 3814^i^, 3815^i^, 3816^i^, 3817^f^, 3818^f^, 3819^i^, 3820^i^**

**3821^i^, 3822^i^, 3823^f^, 3824^i^, 3825^i^, 3826^i^, 3827^t^, 3828^p^, 3829^t^, 3830^i^, 3831^i^**

**3832^i^, 3833^f,i^, 3834^p^, 3835^f^, 3836^p,t^, 3837^i^, 3838^i^, 3839^i^, 3840^i^, 3841^f^, 3842^i^, 3843^f^, 3844^i^, 3845^f^, 3846^t^, 3847^i,f^, 3848^i^, 3849^i^, 3850^f,i^, 3851^i^, 3852^p^, 3853^i^, 3854^i,f^**

**3855^f^, 3856^f,i^, 3857^i^, 3858^i^, 3859^p^, 3860^p^, 3861^i^, 3862^f^, 3863^i^, 3864^i^, 3865^f^, 3866^f^, 3867^i^, 3868^i^**

**3869^t^, 3870^f^, 3871^f^, 3872^f^, 3873^f,i^, 3874^i^**

**110**

**07-1**

**07-2**

**07-3**

**07-4**

**07-5**

**07-6**

**07-7**

**07-8**

**07-9**

**07-10**

**07-11**

**07-12**

**07-13**

**07-14**

**07-15**

**07-16**

**07-17**

**07-18**

**07-19**

**07-20**

**07-21**

**07-22**

**07-23**

**07-24**

**07-25**

**07-26**

**07-27**

**07-28**

**07-29**

**07-30**

**2368^i^, 2369^i^, 2370^i,p^, 2371^p^, 2372^i^, 2373^i^, 2374^t^, 2375^f^, 2376^i^, 2377^i^, 2378^i^, 2379^f^, 2380^p^, 2381^t^, 2382^p^, 2383^i^, 2384^f^, 2385^p^, 2386^i^, 2387^f^**

**2388^i^, 2389^f^, 2390^i^, 2391^f^, 2392^i^, 2393^f^, 2394^t^, 2395^t^, 2396^i^, 2397^i^, 2398^p^, 2399^t^, 2400^i^, 2401^i^**

**2402^p^, 2403^i^, 2404^i^, 2405^i^, 2406^p^, 2407^p^, 2408^p^, 2409^f^**

**2410^t,i^, 2411^f^, 2412^p^, 2413^t^, 2414^i^, 2415^p^, 2416^i^, 2417^p^, 2418^f^, 2419^p^, 2420^i^, 2421^i^, 2422^i^, 2423^i^, 2424^i^, 2425^p^, 2426^f^, 2427^f^, 2428^p^, 2429^f^**

**2430^f,i^, 2431^f^, 2432^i^, 2433^p^, 2434^f^, 2435^p^, 2436^t^, 2437^f^, 2438^i^, 2439^i^, 2440^f^, 2441^i^, 2442^f^, 2443^i^, 2444^f^**

**2445^p^, 2446^f^, 2447^i^, 2448^f^, 2449^p^, 2450^i^, 2451^f^, 2452^i^**

**2453^p^, 2454^i^, 2455^p^, 2456^i^, 2457^i^,^p^, 2458^i^, 2459^i^, 2460^t^**

**2461^i^, 2462^f^, 2463^f^, 2464^i^, 2465^i^, 2466^p^, 2467^f^, 2468^p^, 2469^i^, 2470^f^**

**2471^p^, 2472^i^, 2473^p^, 2474^t,i^**

**2475^i^, 2476^i^, 2477^i^, 2478^i^, 2479^i^, 2480^i^**

**2481^i^, 2482^i^, 2483^i^, 2484^f^, 2485^f^, 2486^f^, 2487^p^**

**2488^i^, 2489^i^**

**2490^f^, 2491^i^, 2492^i^**

**2493^i^, 2494^i^, 2495^i^, 2496^t^, 2497^i^, 2498^f^, 2499^f^, 2500^p^**

**2501^p^, 2502^p^, 2503^f^, 2504^f^, 2505^t^, 2506^f^, 2507^i^**

**2508^i^, 2509^f^, 2510^i^, 2511^f^, 2512^p^, 2513^i^, 2514^f^**

**2515^i^, 2516^p^, 2517^i^, 2518^i^, 2519^t^, 2520^p^, 2521^f^, 2522^p^, 2523^p^**

**2524^i^, 2525^i^, 2526^f^, 2527^t^, 2528^f^, 2529^i^, 2530^i^, 2531^i^, 2532^i^, 2533^p^, 2534^p^**

**2535^p^, 2536^i^, 2537^f^, 2538^p^, 2539^f^, 2540^i^, 2541^i^, 2542^p^, 2543^p^, 2544^i^, 2545^p^, 2546^i^, 2547^i^**

**2548^p^, 2549^i^, 2550^f^, 2551^i^, 2552^i^, 2553^f^, 2554^p^, 2555^i^**

**2556^i^, 2557^p^, 2558^i^, 2559^i^, 2560^i^, 2561^i^, 2562^i^, 2563^i^, 2564^i^**

**2565^p^, 2566^f^, 2567^i^, 2568^t^, 2569^i^, 2570^i^, 2571^f^, 2572^p^**

**2573^p,i^, 2574^i^, 2575^f^, 2576^p^, 2577^f^, 2578^f^, 2579^i^, 2580^i^, 2581^i^, 2582^i^, 2583^i^, 2584^i^, 2585^i^, 2586^i^, 2587^i^, 2588^i^, 2589^i^, 2590^i^, 2591^i^**

**2592^i^, 2593^p^, 2594^i^, 2595^i^, 2596^f^, 2597^t^, 2598^i^, 2599^p^, 2600^i^, 2601^f^, 2602^i^, 2603^i^**

**2604^i^, 2605^p^, 2606^f^, 2607^i^, 2608^i^, 2609^p^, 2610^i^, 1611^i^, 2612^i^, 2613^i^, 2614^f^, 2615^i^, 2616^i^, 2617^i^, 2618^i^, 2619^i^, 2620^p^, 2621^i^**

**2622^p,i^, 2623^i^, 2624^f^, 2625^i^, 2626^i^, 2627^i^**

**2628^i^, 2629^f^, 2630^i^, 2631^p^, 2632^i^, 2633^p^, 2634^t^, 2635^t^, 2636^i^, 2637^i^, 2638^p^, 2639^f^, 2640^p^**

**2641^p^, 2642^t^, 2643^f^, 2644^p^, 2645^p^, 2646^f^**

**2647^i^, 2648^f^, 2649^i^, 2650^t^, 2651^t^, 2652^i^, 2653^t^, 2654^i^, 2655^i^, 2656^i^, 2657^f^, 2658^i^, 2659^i^, 2660^t^, 2661^i^**

**2662^f^**

**2663^f^, 2664^t^, 2665^i^, 2666^i^, 2667^p^, 2668^f^, 2669^i^, 2670^p^, 2671^i^, 2672^f^, 2673^i^**

**08-1**

**08-2**

**08-3**

**08-4**

**08-5**

**08-6**

**08-7**

**08-8**

**08-9**

**08-10**

**08-11**

**08-12**

**08-13**

**08-14**

**08-15**

**08-16**

**08-17**

**08-18**

**08-19**

**08-20**

**08-21**

**08-22**

**08-23**

**08-24**

**08-25**

**08-26**

**08-27**

**08-28**

**08-29**

**2674^i^, 2675^i^, 2676^t^, 2677^t^, 2678^i^, 2679^f^, 2680^f^, 2681^p^, 2682^i^, 2683^i^, 2684^p^, 2685^f^, 2686^i^, 2687^i^, 2688^f^, 2689^i^, 2690^p^**

**2691^i^, 2692^f^, 2693^f,i^, 2694^p^**

**2695^i^, 2696^f^, 2697^i^, 2698^f^, 2699^i^, 2700^i^, 2701^i^, 2702^f^, 2703^f^, 2704^f^, 2705^i^, 2706^i^, 2707^i^, 2708^i^, 2709^p^**

**2710^p^, 2711^i^, 2712^i^, 2713^f^, 2714^p^, 2715^i^, 2716^p^, 2717^p^ 2718^p^, 2719^p^, 2720^i^**

**2721^i^, 2722^f^, 2723^i^, 2724^i^, 2725^i^, 2726^f^, 2727^t^, 2728^i^, 2729^f^, 2730^i^, 2731^i^**

**2732^p,i^, 2733^p,i^, 2743^i^, 2735^p^, 2736^i^, 2773^f^, 7238^i^, 2793^f^, 2704^f,t^, 2741^i^, 2742^i^, 2743^i^**

**2744^f^, 2745^p^, 2746^i^, 2774^i^, 2748^i^**

**2749^f^, 2750^i^, 2751^i^, 2752^f^, 2753^i^, 2754^i^, 2755^i^, 2756^i^, 2757^i^, 2758^i^, 7259^f^, 2760^f^**

**2761^i^, 2762^i^, 2763^i^, 2764^f,i^, 2765^i^, 2766^i^, 2767^t^**

**2768^i^, 2769^p^, 2770^i^, 2771^i^, 2772^i^, 2773^i^**

**2774^i^, 2775^i^, 2776^i^, 2777^p^, 2778^i^, 2779^i^**

**2780^f^, 2781^f^, 2782^i^, 2783^i^, 2784^i^, 2785^i^, 2786^i^, 2787^p^, 2788^i^, 2789^f^**

**2790^i^, 2791^i^, 2792^i^, 2793^i^**

**2794^i^, 2795^i^, 2796^i^**

**2797^p^, 2798^f^, 2799^i^, 2800^i^, 2801^f^, 2802^i^, 2803^i^**

**2804^i^, 2805^i^, 2806^i^, 2807^i^, 2808^i^, 2809^i^, 2810^i^**

**2811^i^, 2812^i^, 2813^i^**

**2814^i^, 2815^i^, 2816^i^, 2817^f^, 2817^f^, 2718^i^, 2819^p^, 2820^i^, 2821^p^**

**2822^p^, 2823^i^, 2824^i^, 2825^p^, 2826^f^**

**2827^i^, 2828^i^, 2829^i^, 2830^p^, 2831^t^, 2832^p^, 2833^i^, 2834^f^, 2835^i^, 2836^i^, 2837^p^**

**2838^t^, 2839^i^, 2840^f^, 2841^i^, 2842^i^, 2843^f^, 2844^f^, 2845^i^, 2846^f,i^, 2847^p,i^, 2848^i^, 2849^f^, 2850^f^, 2851^f^, 2852^i^, 2853^t^**

**2854^i^, 2855^i^, 2856^f^, 22857^f^, 2858^i^, 2859^i^, 2860^f^, 2861^i^, 2862^i^, 2863^i^**

**2864^p^, 2865^p^, 2866^i^, 2867^i^, 2868^i^, 2869^i^, 2870^f^,, 2871^i^, 2872^p^, 2873^i^, 2874^i^**

**8275^i^, 2876^i^, 2877^i^, 2878^i^, 2879^i^, 2880^i^, 2881^f^, 2882^p^, 2883^f^, 2884^f^, 2885^f^, 2886^f^, 2887^i^, 2888^i^, 2889^p^**

**2890^p^, 2891^i^, 2892^i^, 2893^i^, 2894^t^, 2895^f^, 2896^p^, 2897^p,i^, 2898^f^, 2899^p^, 2900^i^, 2901^t^, 2902^t^, 2903^f^, 2904^t^, 2905^i^, 2906^f^, 2907^i^, 2907^i^, 2908^f^, 2809^i^, 2910^i^**

**2911^i^, 2912^i^, 2913^i^, 2913^i^, 2914^i^, 2915^i^, 2916^p^, 1917^i^, 2918^i^, 2919^i^, 2920^i^, 2921^i^**

**2922^i^,^p^, 2923^p^, 2924^f^, 2925^p^, 2926^f^, 2927^f^, 2928^i^, 2929^f^, 2930^i^, 2931^i^, 2932^i^, 2933^f^, 2934^i^, 2935^i,p^, 2936^t,i^, 2937^i^, 2938^f^, 2939^i^, 2940^f^, 2941^i^, 2942^i^, 2943^i^**

**2944^f^, 2945^i^, 2946^t^, 2947^f^, 2948^i^, 2949^p^, 2950^i^, 2951^i^, 2952^f^, 2953^p^, 2954^i^, 2955^i^, 2956^p^, 2957^i^, 2958^p^, 2959^i^, 2960^i,f^, 2961^i^, 2962^f^, 2963^i^, 2964^f^, 2965^p^, 2966^p^, 2967^i^, 2968^t^, 2969^f^**

**2970^i^, 2971^p^**

**2972^t^, 2973^t^, 2974^t^, 2975^f^, 2976^i^**

**09-1**

**09-2**

**09-3**

**09-4**

**09-5**

**09-6**

**09-7**

**09-8**

**09-9**

**09-10**

**09-11**

**09-12**

**09-13**

**09-14**

**09-15**

**09-16**

**09-17**

**09-18**

**09-19**

**09-20**

**09-21**

**09-22**

**09-23**

**2977^i^, 2978^i^, 2979^f^, 2980^p^**

**2981^p^, 2982^i^, 2983^f,p^, 2984^i^, 2985^i^, 2986^i^**

**2987^i^, 2988^i^, 2989^i^, 2990^i^, 2991^t^**

**2992^t^, 2993^i^, 2994^f^, 2995^i^, 2996^p^, 2997^f^, 2998^i^, 2999^i^**

**3000^p^, 30001^i^, 3002^f^, 3003^i^, 3004^f^**

**3005^f^, 3006^i,f^, 3007^i^, 3008^f^, 3009^i^, 3010^i^**

**3011^i,f^, 3012^i^, 3013^i^, 3014^i^, 3015^i^**

**3016^i^, 3017^i^, 3018^f^, 3019^i^, 3020^i^**

**3021^i^, 3022^i^, 3023^i^, 3024^i^, 3025^i^, 3026^i^, 3027^i^, 3028^f^**

**3029^i^, 3030^i^, 3031^f^, 3032^i^, 3033^p^**

**3034^i^, 3035^f^, 3036^i,f^, 3037^i^, 3038^i^, 3039^i^, 3040^i^, 3041^i,f^, 3042^i^**

**3043^i^, 3044^i^, 3045^i^, 3046^i^, 3047^i^, 3048^i^, 3049^i^, 3050^i^, 3051^i^, 3052^i^**

**3053^i^, 3054^t^, 3055^i^, 3056^i^, 3057^t^, 3058^f^, 3059^f^, 3060^p^, 3061^i^**

**3062^p^, 3063^t^, 3064^i^, 3065^i^, 3066^f^, 3067^i^, 3068^i^**

**3069^i^, 3070^i^, 3071^i^, 3072^f^, 3073^i^, 3074^i^, 3075^i^, 3076^t^, 3077^i^, 3078^i^, 3079^i^, 3080^i^, 3081^i^**

**3082^f^, 3083^i^, 3084^i^, 3085^f^, 3086^i^, 3087^i^, 3088^i^, 3089^p^, 3090^t^, 3091^i^, 3092^f^**

**3093^i^, 3094^i^, 3095^i^, 396^i^, 3097^i^, 3098^f^**

**3099^f^, 3100^i^, 3101^f^, 3102^p^, 3103^f^, 3104^i^, 3105^p^, 3106^f^, 3107^f^, 3108^i^**

**3109^i^, 3110^f^, 3111^i^, 3112^i^, 3113^f^, 3114^f^, 3115^f^, 3116^i^, 3117^f^, 3118^p^, 3119^i^, 3120^i^, 3121^t^, 3122^i^, 3123^i^, 3124^t^, 3125^f^**

**3126^f,i^, 3127^i^, 3128^i,f^, 3129^t^, 3130^i^, 3131^i^, 3132^f^, 3133^i^, 3134^i^, 3135^i^, 3136^f^, 3137^f^, 3138^f^, 3139^f^, 3140^p^, 3141^i^**

**3142^i^, 3143^i^, 3144^i^, 3145^f^, 3146^i^, 3147^i^, 3148^p^, 3149^i^, 3150^f,i^, 3151^i^, 3152^p^, 3153^i^, 3154^i^, 3155^f^, 3156^i^, 3157^f^, 3158^i^, 3159^i^**

**3160^i^, 3161^i^, 3162^f,t^, 3163^f^, 3164^i^, 3165^p^, 3166^i^, 3167^f^, 3168^i^, 3169^i^, 3170^i^, 3171^i^, 3172^t^, 3173^p^, 3174^i^, 3175^i^, 3176^f^**

**3177^i^, 3178^i^, 3179^i^, 3180^i^, 3181^f^, 3182^i^, 3183^i^, 3184^i^, 3185^i^, 3186^f^, 3187^t^, 3188^t^, 3189^i^, 3190^f^, 3191^t^**

**10-1**

**10-2**

**10-3**

**10-4**

**10-5**

**10-6**

**10-7**

**10-8**

**10-9**

**10-10**

**10-11**

**10-12**

**10-13**

**10-14**

**10-15**

**10-16**

**10-17**

**10-18**

**10-19**

**10-20**

**10-21**

**10-22**

**10-23**

**3192^i^, 3193^p^, 3194^p^, 3195^i^, 3196^p^, 3197^i^, 3198^i^, 3199^p^, 3200^i^, 3201^i^, 3202^t^**

**3203^i^, 3204^i^, 3205^p^, 3206^i^, 3207^i^, 3208^i^, 3209^i^**

**3210^i^, 3211^f^, 3212^f^, 3213^f^, 3214^i^, 3215^f^, 3216^p^, 3217^p^, 3218^i^**

**3219^i^, 3220^i^, 3221^p^, 3222^p^, 3223^i^**

**3224^i^, 3225^p,i^, 3226^i^, 3227^i^, 3228^i^, 3229^p^**

**3230^p^, 3231^i^, 3232^i^, 3233^f,p^**

**3234^i^, 3235^t^**

**3236^i^, 3237^i^, 3238^i^, 3239^i^, 3240^p^, 3241^p^, 3242^p^**

**3243^t^, 3244^i^, 3245^i^, 3246^f^, 3247^i^**

**3248^i^, 3249^p^, 3250^i^, 3251^i^, 3252^i^, 3253^i^, 3254^f^, 3255^f^**

**3256^i^, 3257^f^, 3258^i^, 3259^p^, 3260^f^, 3261^i^, 3262^f^, 3263^p^, 3264^i^**

**3265^t^, 3266^i^, 3267^i^, 3268^i^, 3269^i^, 3270^i^, 3271^p^, 3272^i^, 3273^p^**

**3274^p^, 3275^i^, 3276^f^, 3277^p^, 3278^i^, 3279^i^, 3280^i^, 3281^f^**

**3282^t^, 3283^p^, 3284^f^, 3285^p^, 3286^i^, 3287^i^, 3288^i^, 3289^i^**

**3290^i^, 3291^p^, 3292^i^, 3293^f^, 3294^i^, 3295^i^, 3296^i^**

**3297^i^, 3298^p^, 3299^i^, 3300^i^, 3301^p^, 3302^t^, 3303^i^, 3304^f^, 3305^p^, 3306^i,t^, 3307^f^, 3308^i,f^, 3309^i^, 3310^t^, 3311^i^**

**3312^p^, 3313^i^, 3314^i^, 3315^i^, 3316^f^, 3317^i^, 3318^f^, 3319^f^, 3320^i^, 3321^i^, 3322^p^, 3323^f^, 3324^f^, 3325^i,p^, 3326^f^**

**3327^i^, 3328^f^, 3329^i^, 3330^i^, 3331^i^, 3333^i^, 3334^f^, 3335^i^, 3336^i^, 3337^i^, 3338^t^, 3339^i^, 3340^f^, 3341^i^, 3342^f^, 3343^p^**

**3344^f^, 3345^i^, 3346^p^, 3347^i^, 3348^f^, 3349^p^, 3350^t^, 3351^f^, 3352^f^**

**3353^i^, 3354^p^, 3355^p^, 3356^i^, 3357^p^, 3358^t^, 3359^p^, 3360^t^, 3361^p^, 3362^t^, 3363^t^, 3364^i^, 3365^p^, 3366^i^, 3367^p^, 3368^i^**

**3369^i^, 3370^i^**

**3371^f^, 3172^f^, 3173^i^, 3374^i^, 3375^i^, 3376^p^, 3377^p^, 3378^t^, 3379^i^, 3380^f^, 3381^i^, 3382^p^, 3383^i^, 3384^p^, 3385^i^**

**3386^i^, 3387^i^, 3388^i^, 3389^p^, 3390^p^, 3391^i^, 3392^i^, 3393^f^, 3394^i^, 3395^i^, 3396^i^, 3397^i^, 3398^f^, 3399^p^, 3400^f^, 3401^p^, 3402^f^, 3403^i^**

**3404^i^, 3405^i^, 3405^i^, 3406^i^, 3407^f^, 3408^i^, 3409^i^, 3410^i^, 3411^i^, 3412^f^, 3413^i^, 3414^i^, 3415^f^, 3416^i^, 3419^i^**

**3420^p^, 3421^p^, 3422^p^, 3423^i^, 3424^t^, 3425^i^, 3426^i^, 3427^f^, 3428^p^, 3429^f^, 3430^i^, 3431^i^, 3432^p^, 3433^i^, 3443^i^**

**3435^i^, 3436^i^, 3437^i^, 3438^f^, 3439^f,p^, 3440^i^**

**3441^i^, 3424^i^, 3443^p^, 3444^i^, 3454^f^, 3446^t^, 3447^i^, 3448^i^, 3449^i^, 3450^f^, 3451^f^, 3452^i^, 3453^i^, 3454^p^, 3455^f^**

**3456^i^, 3457^f^, 3458^i^, 4359^i^, 3460^i^, 4361^i^**

**3462^i^, 3463^f^, 3464^i^, 3465^t^, 3466^p^, 3467^i^**

**3468^f^, 3469^i^, 3470^i^, 3471^p,i^, 3472^f^**

**3473^p^, 3474^i^, 3475^f^, 3476^i^**

**3477^i^, 3478^i^, 3479^p^, 3480^p^**

**3481^i^, 3482^i^, 3483^i^, 3484^i^**

**3485^p^, 3486^i^, 3487^p^, 3488^p^, 3489^i^, 3490^i^, 3491^i^, 3492^p^**

**3493^p^, 3494^i^, 3495^i^, 3496^p^, 3497^i^**

**3498^i^, 3499^i^, 3499^i^, 3500^i^, 3501^i^, 3502^i^**

**3503^p^, 3504^i^, 3505^i^, 3506^i^, 3507^i^**

**3508^t^, 3509^p^, 3510^i^, 3511^i^, 3512^t^, 3513^i^**

**3514^i^, 3515^i^, 3516^f^**

**3517^p^, 3518^i^, 3519^p^, 3520^i^**

**3521^f^, 3522^i^, 3523^t^, 3524^f^, 3525^i^, 3526^p^, 3527^i^**

**3528^t^, 3529^i^, 3530^i^, 3531^i^, 3532^p^, 3533^f^, 3534^f^, 3535^i^, 3536^i^, 3537^i^, 3538^i^, 3539^i^**

**3540^f^, 3541^p^, 3542^i^, 3543^i^, 3544^f^, 3545^f^, 3546^i^**

**3547^p^, 3548^p^, 3549^f^, 3550^f^, 3551^f^, 3552^p^, 3553^f^, 3554^i^, 3555^p^**

**3556^f^, 3557^f^, 3558^i^, 3559^f^, 3560^i^**

**3561^i^, 3562^i^, 3563^i^, 3564^i^, 3565^i^, 3566^i^, 3567^i^**

**3568^i^, 3569^i^, 3570^i^, 3571^i^, 3572^i,t^, 3573^f^**

**3574^i^, 3575^i^, 3576^f^, 3577^i^, 3578^i^**

**3579^i^, 3580^i^, 3581^i^, 3582^i^, 3583^i^, 3584^i^, 3585^i^, 3586^i^**

**3587^i^, 3588^i^, 3589^i^, 3590^i^, 3591^i^, 3592^i^, 3593^i^, 3594^i^, 3595^i^, 3596^i^, 3597^i^, 3598^i^, 3599^i^, 3600^i^, 3601^i^**

**3602^t^, 3603^f^, 3604^t^, 3605^i^, 3606^i^, 3607^f^, 3608^i^, 3609^i^, 3610^f^, 3611^f^**

**11-1**

**11-2**

**11-3**

**11-4**

**11-5**

**11-6**

**11-7**

**11-8**

**11-9**

**11-10**

**11-11**

**11-12**

**11-13**

**11-14**

**11-15**

**11-16**

**11-17**

**11-18**

**11-19**

**11-20**

**11-21**

**11-22**

**11-23**

**11-24**

**11-25**

**11-26**

**11-27**

**11-28**

**11-29**

**Additional file 5: Physical bin map of rice using 4,288 class I GNMS markers with an average density of 100.7 kb. The bin (1Mb interval sized) numbers with their chromosomal specifications are indicated on the left side. The identity of the markers are given on the right side that corresponds to the marker IDs ‘RGNMS’ and gene locus IDs of TIGR as given in the Additional file 3. The GNMS markers designed from the promoter (p), 5’UTR (f), intron (i) and 3’UTR (t) sequences of rice genes are indicated. Abbreviation ‘RGNMS’ has been used for Rice Genic Non-coding MicroSatellites. The size of the chromosome is based on the TIGR rice pseudomolecule 5.0 database (**[**http://www.tigr.org/tdb/e2k1/osa1**](http://www.tigr.org/tdb/e2k1/osa1)**) dated 24^th^ Jan’2007.**
