# Supplementary material for: Genic non-coding microsatellites in the rice genome: characterization, marker design and use in assessing genetic and evolutionary relationships among domesticated groups
Source: BMC Genomics. 2009 Mar 31;10:140. doi: 10.1186/1471-2164-10-140 (PMC2680414; doi:10.1186/1471-2164-10-140)
Supplement: Additional file 8 — Origin, distribution and polymorphic potential of 30 GNMS markers and four markers from the CDS, in a set of 18 rice genotypes. [file 1471-2164-10-140-S8.doc]

**Additional file 8:** **Origin, distribution and polymorphic potential of 30 GNMS and four CDS derived markers**

| **Genes** | **GNMS marker origin** | **Rice chromosome - Gene locus ID** | **GNMS repeat-motifs** | **Number of alleles** | **PIC value** | **Unique allele** |
| --- | --- | --- | --- | --- | --- | --- |
| Heat shock protein | Promoter | 3->LOC_Os03g25120.1|11973.m07855 | (TA)6 | 2 | 0.51 | - |
| β-galactosidase | Promoter | 3->LOC_Os03g15020.2|11973.m34822 | (GA)13 | 6 | 0.81 | Short grain aromatics & *japonica* |
| Sucrose synthase | Promoter | 3->LOC_Os03g28330.1|11973.m08153 | (GAGGA)5 | 3 | 0.63 | Non-aromatic *indica* |
| PPR protein | Promoter | 3->LOC_Os03g56400.1|11973.m10549 | (CGC)8 | 3 | 0.68 | Non-aromatic *indica* |
| ATP synthase | Promoter | 3->LOC_Os03g17070.1|11973.m07139 | (GA)20 | 6 | 0.81 | *japonica* varieties |
| Sucrose transporter | Promoter | 2->LOC_Os02g36700.1|11972.m08757 | (TA)26 | 2 | 0.55 | Short grain aromatics & *japonica* |
| Ubiquitin | Promoter | 2->LOC_Os02g02830.2|11972.m33517 | (TAT)26 | 5 | 0.66 | *japonica* varieties |
| F box domain | Promoter | 2->LOC_Os02g21260.1|11972.m07366 | (AT)43 | 3 | 0.59 | - |
| RUBP | 5’UTR | 3->LOC_Os03g07300.1|11973.m06244 | (AG)7 | 4 | 0.71 | - |
| Transcription factors | 5’UTR | 1->LOC_Os01g17260.1|11971.m08319 | (CCCCT)6 | 2 | 0.20 | *japonica* varieties |
| Ras-GTP binding protein | 5’UTR | 2->LOC_Os02g02840.1|11972.m05634 | (GA)21 | 6 | 0.79 | - |
| Wuschel homeobox | 5’UTR | 1->LOC_Os01g60270.1|11971.m12157 | (GA)26 | 5 | 0.72 | Swarna |
| Protein kinase | 5’UTR | 1->LOC_Os12g23940.1|11982.m06229 | (GAC)7 | 4 | 0.74 | Non-aromatic *indica* & aromatics |
| Seryl tRNA synthase | 5’UTR | 1->LOC_Os01g37837.1|11971.m43477 | (CGC)7 | 3 | 0.56 | - |
| Protein kinase | CDS | 3->LOC_Os03g47470.1|11973.m09757 | (CCT)5 | 2 | 0.10 | - |
| ATPase | CDS | 3->LOC_Os03g58800.1|11973.m10783 | (GCG)8 | 2 | 0.10 | *japonica* varieties |
| Phospholipase | CDS | 3->LOC_Os03g02740.1|11973.m05814 | (GAA)10 | 2 | 0.10 | - |
| LRR protein | CDS | 1->LOC_Os03g58110.1|11973.m10723 | (CGC)7 | 2 | 0.10 | - |
| Protein kinase | Intron | 3->LOC_Os01g01410.1|11971.m06787 | (TCT)6 | 3 | 0.53 | *japonica* varieties |
| O-acetyl transferase | Intron | 1->LOC_Os01g44040.1|11971.m10663 | (TG)9 | 3 | 0.56 | *japonica* varieties |
| NBS-LRR disease resistance | Intron | 1->LOC_Os11g11810.1|11981.m05373 | (AAAT)5 | 4 | 0.68 | Aromatics & Non-aromatic *indica* |
| PPR | Intron | 1->LOC_Os01g63400.1|11971.m12456 | (CA)15 | 5 | 0.73 | *japonica* varieties |
| Adenosine mono-phosphate | Intron | 1->LOC_Os01g24030.2|11971.m43003 | (GAG)8 | 5 | 0.76 | *japonica* varieties |
| Glucose-1-phosphate | Intron | 1->LOC_Os01g44220.1|11971.m10680 | (AT)13 | 5 | 0.74 | *japonica* varieties |
| PhospholipaseD | Intron | 1->LOC_Os01g20860.1|11971.m08570 | (AAG)7 | 6 | 0.80 | *-* |
| Ubiquitin | Intron | 3->LOC_Os03g50440.1|11973.m10036 | (GA)25 | 7 | 0.86 | *japonica* varieties |
| Peroxidase | Intron | 1->LOC_Os01g36240.1|11971.m09920 | (TA)43 | 6 | 0.81 | *japonica* varieties |
| Ribosomal protein S35 | Intron | 1->LOC_Os01g01060.1|11971.m06753 | (AT)40 | 8 | 0.84 | *japonica* varieties |
| Serine carboxy peptidase | Intron | 3->LOC_Os03g27480.1|11973.m08075 | (AAT)29 | 4 | 0.70 | - |
| Β-ketoacyl CoA synthase | 3’UTR | 3->LOC_Os03g26530.1|11973.m07989 | (AC)7 | 4 | 0.57 | Aromatics & *japonica* |
| Plastocyanin | 3’UTR | 3->LOC_Os02g49350.1|11972.m09969 | (AAG)6 | 4 | 0.68 | - |
| ATP-NAD kinase | 3’UTR | 1->LOC_Os01g72690.1|11971.m13303 | (CATATA)5 | 4 | 0.51 | Non-aromatic *indica* |
| Lipase | 3’UTR | 5->LOC_Os05g11950.1|11975.m05662 | (GA)33 | 5 | 0.73 | - |
| Cytochrome P450 | 3’UTR | 3->LOC_Os01g10040.2|11971.m42908 | (TA)30 | 3 | 0.43 | Non-aromatic *indica* |
