# Supplementary material for: Genic non-coding microsatellites in the rice genome: characterization, marker design and use in assessing genetic and evolutionary relationships among domesticated groups
Source: BMC Genomics. 2009 Mar 31;10:140. doi: 10.1186/1471-2164-10-140 (PMC2680414; doi:10.1186/1471-2164-10-140)
Supplement: Additional file 9 — Alignment showing the presence of class I GNMS repeat-motifs across 18 genotypes of rice. [file 1471-2164-10-140-S9.doc]

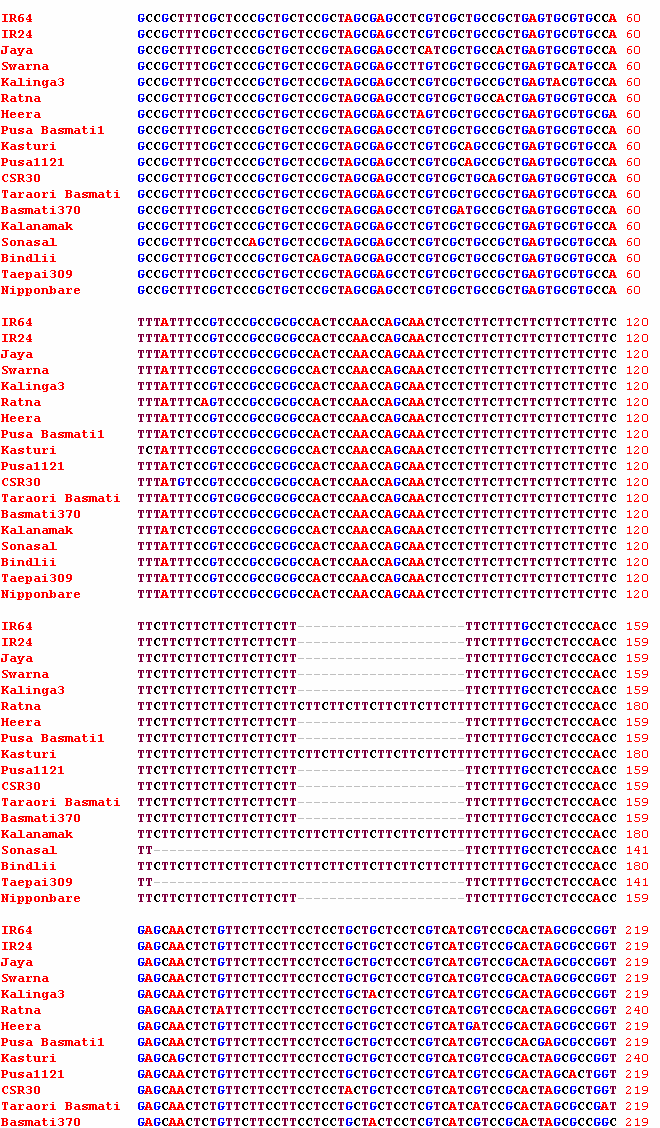


**Additional file 9: Alignment of class I (TCT)_n_ GNMS repeat-motif region present in the promoter sequences of β-galactosidase gene of 18 rice genotypes. The genotypes Sonasal and Taepai309 contained (TCT)_7_ GNMS motifs, whereas the expansion of repeat-motif to (TCT)_13_ is observed in the genotypes IR64, IR24, Jaya, Swarna, Kalinga3, Heera, Pusa Basmati1, Pusa1121, CSR30, Taraori Basmati, Basmati370 and Nipponbare, and to (TCT)_20_ in Ratna, Kasturi, Kalanamak and Bindli, respectively.**
