# Supplementary material for: Genic non-coding microsatellites in the rice genome: characterization, marker design and use in assessing genetic and evolutionary relationships among domesticated groups
Source: BMC Genomics. 2009 Mar 31;10:140. doi: 10.1186/1471-2164-10-140 (PMC2680414; doi:10.1186/1471-2164-10-140)
Supplement: Additional file 10 — Genetic relationships among 18 indica and japonica domesticated rice cultivars revealed by 56 rice GNMS markers. [file 1471-2164-10-140-S10.doc]

**Additional file 10: Genetic relationships among 18 rice genotypes revealed by 56 rice GNMS markers using Nei and Li’s similarity coefficient**

| **Genetic relationships** | **Similarity coefficient** | | |
| --- | --- | --- | --- |
|  | **Minimum** | **Maximum** | **Average** |
| Within *indica* varieties | 0.47  (Swarna & IR24) | 0.69  (Heera & Kalinga3) | 0.58 |
| Within short-grained aromatics | 0.57  (Kalanamak & Sonasal) | 0.68  (Bindli & Kalanamak) | 0.64 |
| Within long-grained aromatics | 0.29  (Taraori Basmati & Kasturi) | 0.58  (Pusa Basmati1 & Pusa1121) | 0.42 |
| Within traditional Basmati varieties | - | - | 0.56 |
| Within evolved Basmati varieties | 0.34  (CSR30 & Kasturi) | 0.58  (Pusa Basmati1 & Pusa1121) | 0.40 |
| Between traditional Basmati and evolved Basmati varieties | 0.29  (Taraori Basmati & Kasturi) | 0.54  (Basmati370 & Pusa Basmati1) | 0.42 |
| Within *japonica* cultivars | - | - | 0.74 |
| Between *indica* and long-grained aromatics | 0.24  (IR64 & Pusa 1121) | 0.33  (Kalinga 3 & Pusa Basmati1) | 0.28 |
| Between *indica* and short-grained aromatics | 0.26  (Swarna & Sonasal) | 0.40  (IR24 & Bindli) | 0.32 |
| Between *indica* and *japonica* | 0.15  (IR64 & Nipponbare) | 0.17  (Jaya & Taepai 309) | 0.13 |
| Between short-grained and long-grained aromatics | 0.24  (Pusa Basmati1 & Sonasal) | 0.34  (Kalanamak & Kasturi) | 0.30 |
| Between short-grained aromatics and *japonica* | 0.17  (Kalanamak & Nipponbare) | 0.29  (Sonasal & Nipponbare) | 0.22 |
| Between long-grained aromatics and *japonica* | 0.17  (Pusa1121 & Nipponbare) | 0.32  (Basmati 370 & Nipponbare) | 0.25 |
