# Supplementary material for: Genic non-coding microsatellites in the rice genome: characterization, marker design and use in assessing genetic and evolutionary relationships among domesticated groups
Source: BMC Genomics. 2009 Mar 31;10:140. doi: 10.1186/1471-2164-10-140 (PMC2680414; doi:10.1186/1471-2164-10-140)
Supplement: Additional file 15 — Positional distribution of orthologous and paralogous CNMS in promoter sequences of rice genes. [file 1471-2164-10-140-S15.doc]

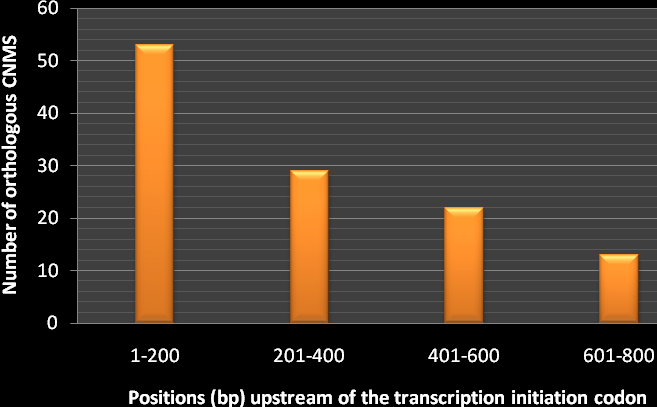

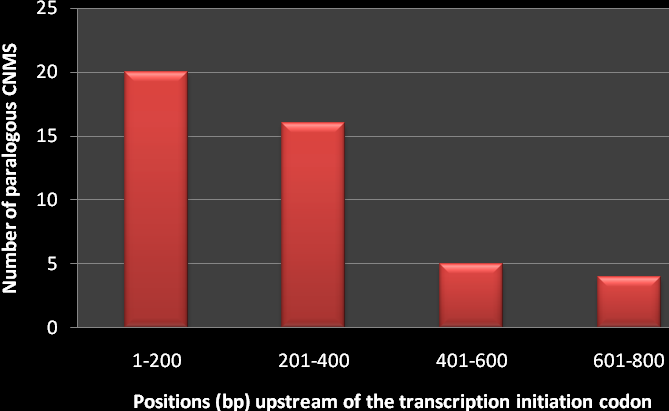


**A**

**B**

**Additional file 15: Positional distribution of 112 orthologous (A) and 45 paralogous (B) CNMS in the promoter sequences of rice genes**
