# Supplementary material for: Genic non-coding microsatellites in the rice genome: characterization, marker design and use in assessing genetic and evolutionary relationships among domesticated groups
Source: BMC Genomics. 2009 Mar 31;10:140. doi: 10.1186/1471-2164-10-140 (PMC2680414; doi:10.1186/1471-2164-10-140)
Supplement: Additional file 16 — CNMS marker based comparative physical mapping showing colinearity between rice chromosome 1 and homeologous chromosomes of four other cereal species and Arabidopsis thaliana. [file 1471-2164-10-140-S16.doc]

**01-1**

**01-2**

**01-3**

**01-4**

**01-5**

**01-6**

**01-7**

**01-8**

**01-9**

**01-10**

**01-11**

**01-12**

**01-13**

**01-14**

**01-15**

**01-16**

**01-17**

**01-18**

**01-19**

**01-20**

**01-21**

**01-22**

**01-23**

**01-24**

**01-25**

**01-26**

**01-27**

**01-28**

**01-29**

**01-30**

**01-31**

**01-32**

**01-33**

**01-34**

**01-35**

**01-36**

**01-37**

**01-38**

**01-39**

**01-40**

**01-41**

**01-42**

**01-43**

**01-44**

**5^p^, 7^p^, 9^p^, 15^p^**

**22^p^, 26^p^, 27^p^**

**29^p^**

**36^p^, 41^p^, 44^p^, 46^p^**

**50^p^, 51^p^, 56^p^, 60^p^**

**63^p^, 69^p^**

**75^p^, 76^p^**

**83^p^, 85^p^, 91^p^, 94^p^,**

**120^p^**

**127^p^**

**136^p^, 142^p^**

**145^p^**

**156^p^**

**162^p^, 165^p^, 170^p^**

**178^p^**

**186^p^, 187^p^**

**191^p^, 192^p^**

**197^p^, 198^p^, 202^p^**

**206^p^, 207^p^**

**213^p^, 215^p^, 218^p^**

**221^p^, 230^p^**

**232^p^, 233^p^**

**247^p^**

**257^p^**

**264^p^**

**280^p^, 281^p^, 283^p^**

**295^p^**

**296^p^, 302^p^, 304 ^p^, 306^p^**

**309^p^, 310^p^, 313^p^, 316^p^, 317^p^**

**320^p^, 328^p^, 329^p^, 334^p^**

**335^p^, 340^p^**

**358^p^**

**359^p^**

**375^p^, 382^p^**

**401^p^, 406^p^, 407^p^, 408^p^**

**414 ^p^, 415^p^, 425^p^**

**436^p^**

**440^p^, 441^p^, 442^p^, 443^p^, 451^p^**

**461^p^**

**470^p^, 471^p^**

**484^p^**

**Bins**

**Bins**

**Bins**

**3.00**

**3.01**

**3.02**

**3.03**

**3.04**

**3.05**

**3.06**

**3.07**

**3.08**

**3.09**

**3.10**

**RCh1**

**WCh3**

**(Consensus)**

**3S-0.78-1.00**

**3S-0.57-0.78**

**3S-0.55-0.57**

**3S-0.45-0.55**

**3S-0.33-0.45**

**3S-0.24-0.33**

**3S-0.23-0.24**

**C-3S-0.23**

**C-3L-0.22**

**3L-0.22-0.27**

**3L-0.27-0.42**

**3L-0.42-0.50**

**3L-0.50-0.63**

**3L-0.63-0.78**

**3L-0.78-0.81**

**3L-0.81-1.00**

**MCh3**

**1**

**2**

**3**

**4**

**5**

**6**

**7**

**8**

**9**

**10**

**11**

**12**

**13**

**14**

**15**

**16**

**17**

**18**

**19**

**20**

**21**

**22**

**23**

**AtCh3**

**1**

**2**

**3**

**4**

**5**

**6**

**7**

**8**

**9**

**10**

**11**

**12**

**13**

**14**

**15**

**16**

**BCh3 (3H)**

**SCh3**

**1**

**2**

**3**

**4**

**5**

**6**

**7**

**8**

**9**

**10**

**11**

**12**

**13**

**14**

**15**

**16**

**17**

**18**

**19**

**20**

**Additional file 16: CNMS marker based comparative physical map showing collinearity between the rice chromosome 1 and homeologous chromosomes of four other cereal species and *Arabidopsis***
