# Supplementary material for: Genic non-coding microsatellites in the rice genome: characterization, marker design and use in assessing genetic and evolutionary relationships among domesticated groups
Source: BMC Genomics. 2009 Mar 31;10:140. doi: 10.1186/1471-2164-10-140 (PMC2680414; doi:10.1186/1471-2164-10-140)
Supplement: Additional file 17 — List of 18 rice genotypes used for studying the polymorphic potential of 60 GNMS markers and their comparison with 15 microsatellite markers designed from CDS. [file 1471-2164-10-140-S17.doc]

Additional file 17: List of 18 rice genotypes used for studying the polymorphic potential of 60 GNMS markers

| Sl. No. | Species used under study |
| --- | --- |
|  | *indica* |
| 1 | IR-64 |
| 2 | IR-24 |
| 3 | Jaya |
| 4 | Swarna |
| 5 | Kalinga3 |
| 6 | Ratna |
| 7 | Heera |
|  | Long-grained aromatics |
| 8 | Pusa Basmati1 |
| 9 | Kasturi |
| 10 | Pusa1121 |
| 11 | CSR30 |
| 12 | Taraori Basmati |
| 13 | Basmati370 |
|  | Short-grained aromatics |
| 14 | Kalanamak |
| 15 | Sonasal |
| 16 | Bindlii |
|  | *japonica* |
| 17 | Taepai309 |
| 18 | Nipponbare |
